# Traditional Chinese medicine lowering lipid levels and cardiovascular events across baseline lipid levels among coronary heart disease: a meta-analysis of randomized controlled trials

**DOI:** 10.3389/fcvm.2024.1407536

**Published:** 2024-07-11

**Authors:** Lihua Xie, Jia Liu, Xiaochi Wang, Birong Liu, Jiaqi Li, Jingen Li, Huanlin Wu

**Affiliations:** ^1^ Department of Cardiology, Dongzhimen Hospital, Beijing University of Chinese Medicine, Beijing, China; ^2^ Graduate School, Beijing University of Chinese Medicine, Beijing, China; ^3^ Department of Cardiology, Xi'an Hospital of Traditional Chinese Medicine, Xi'an, Shanxi, China

**Keywords:** Chinese herbal medicine, coronary heart disease, cardiovascular events, lipids profile, meta-analysis

## Abstract

**Background:**

Dyslipidemia is a critical driver in the development of coronary heart disease (CHD), which further exacerbates the risk of major adverse cardiovascular events (MACEs). Chinese herbal medicine (CHM) plays an important role in the regulation of lipid levels and improvement of prognosis. However, few systematic reviews report whether the efficacy of CHM therapy for regulating lipid levels and lowering cardiovascular events is associated with baseline lipid levels.

**Methods:**

Randomized controlled trials assessing efficacy of CHM for lipid profiles and MACEs among patients with CHD were searched in six databases. Two authors independently extracted studies according to a predesigned form. Cochrane risk of bias tool and Grading of Recommendations Assessment, Development, and Evaluation system were used to assess the methodological quality of the included studies. The primary outcomes were blood lipid levels and MACEs including cardiovascular mortality, non-fatal myocardial infarction, revascularization, angina pectoris, heart failure, and non-fatal stroke across baseline lipid levels. The secondary outcomes were individual components of the primary outcomes.

**Results:**

A total of 23 trials with 7,316 participants were included in this study. Altogether 377 cardiovascular events occurred in 3,670 patients in the CHM group, while 717 events occurred in 3,646 patients in the Western medicine–alone group. Compared with the Western medicine alone, additional CHM significantly lowered low-density lipoprotein cholesterol (LDL-C) [MD = −0.46, 95% CI (−0.60 to −0.32), *P *< 0.00001, *I*^2^ = 96%]. The risk reduction in MACEs associated with CHM vs. Western medicine therapy was 0.52 [95% CI (0.47–0.58), *P *< 0.00001, *I*^2^* *= 0%], but varied by baseline LDL-C level (*P *= 0.03 for interaction). Triglycerides (TG) level was also significantly lowered by additional CHM vs. Western medicine alone [MD = −0.27, 95% CI (−0.34 to −0.20), *P *< 0.00001, *I*^2^ = 81%], and risk reduction for MACEs also varied with baseline TG, with greater risk reduction in higher baseline TG subgroups (*P *= 0.03 for interaction). Similar results were observed with total cholesterol and high-density lipoprotein cholesterol.

**Conclusion:**

Compared with Western medicine alone, additional CHM was associated with lower risk of cardiovascular events and improvement of lipid profiles. Risk reduction for cardiovascular events was associated with baseline LDL-C and TG levels.

**Systematic Review Registration:**

https://www.crd.york.ac.uk/PROSPERO, identifier CRD42023425791.

## 1 Introduction

Coronary heart disease (CHD) is a major contributor to the global disease burden because of its high morbidity and mortality (1). An epidemiological study has shown that the population of this disease 42 were 197 million, and ultimately led to 9.14 million deaths in 2019, a 50.4% increase from 1990 (2). Lipid metabolism disorder has been considered as the pathological basis of atherosclerosis, and thus CHD, and also a risk predictor for prognosis in patients with CHD (3, 4). Evidence has proven that a high cholesterol level is a causal factor for cardiovascular events, and active pharmacological therapy for hypercholesterolemia could actually alleviate the pathologies of CHD (5, 6). Recent data from meta-analysis showed a 23% relative reduction in cardiovascular risk per mmol/L reduction in low-density lipoprotein cholesterol (LDL-C) (7), and triglycerides (TG) lowering is associated with about half the cardiovascular risk reduction per mmol/L (8).

Evidence has shown that Western medicine mainly reduces the occurrence of cardiovascular events by lowering the level of LDL-C in patients with CHD (9, 10), and by using antiplatelets regularly and controlling other risk factors to relieve atherosclerosis. Although the current treatments for CHD have been consummate, and are constantly being updated with the deepening research, there are still residual cardiovascular risks (11). Therefore, novel treatments that can control blood lipids, stabilize arterial plaque, diminish adverse effects, and reduce cardiovascular events are urgently needed. Chinese herbal medicine (CHM) is a holistic approach characterized by the overall adjustment and differentiation of syndromes, and has a long history in the treatment of CHD. Many clinical trials have confirmed that CHM plays an important role in cardioprotective function and regulation of lipid levels (12, 13). Simultaneously, the cardiovascular outcome benefits were observed (14). Although there have been meta-analysis confirming that CHM could reduce blood lipids and cardiovascular events, few studies report whether the risk reduction of cardiovascular events is associated with baseline lipid levels and the magnitude of lipid reduction. Thus, the purpose of this systematic review was to elucidate the efficacy of CHM therapy for regulating lipid levels and lowering cardiovascular events across baseline lipid levels and magnitude of lipid reduction among CHD.

## 2 Methods

### 2.1 Study registration

This systematic review was conducted and reported according to the PRISMA (Preferred Reporting Items for Systematic Reviews and Meta-Analyses Guideline) statement (15). The protocol was registered in PROSPERO (https://www.crd.york.ac.uk/PROSPERO), and the registration number is CRD42023425791.

### 2.2 Search strategy and study selection

Published articles searches were performed by two independent authors (LX and JL) using the following databases: PubMed, Web of science, Cochrane library, China National Knowledge Infrastructure (CNKI), Wanfang Database, and Chinese Scientific Journals Database (VIP), up to 18 December 2022. Search terms such as “coronary heart disease”, “myocardial infarction”, “traditional Chinese medicine”, “lipid”, “LDL-C”, “HDL-C”, “cholesterol”, “triglyceride”, “randomized controlled trial”, and their synonyms were applied in the search strategy. Detailed search strategies can be found in the Supplementary Material S1. The frequency statistics of single CHM was conducted to determine the commonly used drugs.

### 2.3 Inclusion and exclusion criteria

The main inclusion criteria were as follows: (1) All participants were diagnosed with CHD; (2) Randomized controlled clinical trials included at least 30 patients in each group; (3) Treatments including conventional Western medicine (i.e., antiplatelet, stable plaque, improve ventricular remodeling, etc.) were used in both the control and intervention group, and oral Chinese medicine was administered in the intervention group; and (4) Outcomes of interest involved blood lipid parameters and cardiovascular events.

The exclusion criteria were as follows: (1) Oral Chinese medicine was used in the control group or non-oral Chinese medicine was used in the CHM group; (2) Non-clinical research, review, meta-analysis, and non-core journals; (3) Studies with incomplete information.

### 2.4 Study selection and data extraction

The retrieved studies were extracted by two authors (LX and XW) independently following the inclusion and exclusion criteria and included the following details: title, first author, document source, year of publication, sample size, age, gender, disease, interventions, course of treatment, follow-up time, and outcome indicators. Any disagreements were settled through consultation with the third author (BL).

### 2.5 Outcomes

The primary outcomes were blood lipid parameters [LDL-C, TG, total cholesterol (TC), and high-density lipoprotein (HDL)] and major adverse cardiovascular events (MACEs) including cardiovascular mortality, non-fatal myocardial infarction, non-fatal stroke, angina pectoris, heart failure, and revascularization (percutaneous coronary intervention or coronary artery bypass graft surgery). The secondary outcomes were individual components of the primary outcomes.

### 2.6 Quality assessment

The methodological quality of the selected studies was assessed by two authors (LXH and JJ) separately according to the seven-item checklist of the Cochrane risk of bias tool (16). The studies that included sufficient domain information were regarded as low risk, while studies with inadequate information were considered as unclear risk. In addition, evaluations were also conducted based on the loss to follow-up and comparability between groups. Furthermore, the Grading of Recommendations, Assessment, Development and Evaluation (GRADE) approach was used to assess the quality of evidence for primary outcomes (17). We followed the GRADE handbook to conduct the quality assessment, and the GRADEpro Guideline Development Tool (GDT) was used to generate the results (18). Any disagreements were settled through consultation with the third author (JiaL).

### 2.7 Data analysis and synthesis

Statistical analyses were conducted using Review Manager (RevMan) version 5.4 (The Cochrane Collaboration, 2020). The rate ratios (RR) and weighted mean difference (WMD) were applied to analyze the dichotomous data and continuous data. The heterogeneity across the included studies was measured by *I*^2^ statistic and Cochran's Q-test. *I*^2^ > 50% or *P* < 0.05 was considered as having statistically significant heterogeneity.

### 2.8 Publication bias, subgroup, and sensitivity analysis

Publication bias was detected by funnel plots and Egger's test. To explore the potential source of heterogeneity, the meta-regression analyses, subgroup (Age: <60, ≥60; Statins: used statins, not used statins; course of treatment: <6, 6–12, ≥12 months; type of disease: post-PCI, CCS, ACS) analyses, and sensitivity analysis by omitting one trial at a time were performed as there was significant heterogeneity.

## 3 Results

### 3.1 Literature search

**Figure 1:**
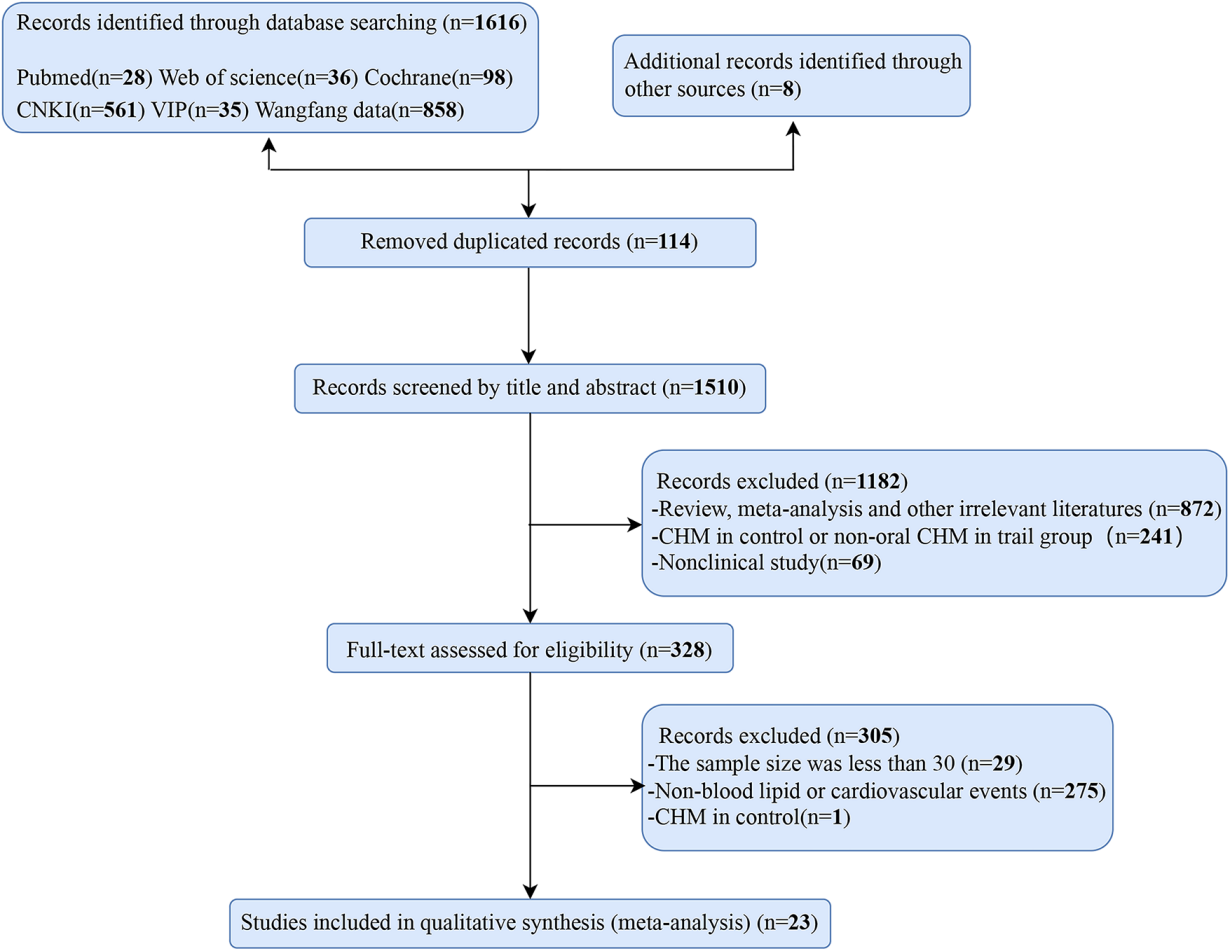
Flow diagram of articles selection procedures.

A total of 1,624 records were initially identified, of which 114 were duplicated. Subsequently, 1,182 were excluded after carefully screening titles and abstracts because they were (1) review, meta-analysis, meeting abstract, and irrelevant literatures; (2) CHM in control or non-oral CHM in trial group; and (3) non-clinical study. Further, 305 were excluded after reviewing the full text because: (1) the sample size was less than 30; (2) no lipid parameters and cardiovascular events were recorded; and (3) CHM was used in the control group. Ultimately, 23 studies were identified to be eligible and subsumed in the meta-analysis (Figure 1).

### 3.2 Study characteristics

Of the selected studies, 22 were published in Chinese and 1 in English between 2004 and 2022. The baselines of all the studies were comparable. A total of 7,316 participants were included, with 3,670 participants in the CHM group and 3,646 in the control group. The subjects of CHD included stable angina (SA), unstable angina (UA), acute coronary syndrome (ACS), myocardial infarction (MI), non-ST segment elevation myocardial infarction (NSTEMI), ST segment elevation myocardial infarction (STEMI), variant angina pectoris (VAP), post-percutaneous coronary intervention (post-PCI), and borderline lesion CHD. Twenty-two out of the 23 trials were conducted for comparisons of CHM combined conventional treatment therapy vs. a conventional treatment alone (19–40), one trial performed the comparisons of CHM therapy vs. a placebo (41). The CHM in the intervention group was administered orally (decoction, tablets, capsules, granules, or pills). The duration of follow-up varied from 15 days to 4.5 years. The levels of LDL-C were reported in 23 studies, the levels of HDL-C in 17 studies, the levels of TG in 20 studies, and the levels of TC in 20 studies. The overall characteristics are provided in Table 1.

**Table 1:** Characteristics of the included studies.

Study	Disease	*N* (male/female), mean age (years)		Mean age (years)		Interventions		Basic treatment	Course of treatment (duration of follow-up)	Baseline LDL-C mean (SD), mmol/L	Baseline HDL-C mean (SD), mmol/L	Baseline TC mean (SD), mmol/L	Baseline TG mean (SD), mmol/L	MACEs
		T	C	T	C	T	C							
Xu et al. (19)	Post-PCI	100 (52/48)	100 (51/49)	59.76 ± 10.43	59.36 ± 10.13	Shexiang Baoxin pill	*N*	① ②	3 months (12 months)	2.71 ± 0.65	1.19 ± 0.21	5.72 ± 1.02	1.71 ± 0.43	AP, NFAMI, CD
Li et al. (20)	UA	118 (61/57)	110 (55/55)	60.1 ± 10.5	60.5 ± 9.5	Wenxin decoction	*N*	① ② ③ ⑤ ⑥	60 days (60 days)	4.25 ± 1.24	—	—	—	NFAMI, RV, NFS, CD
Yang et al. (21)	SA	100 (58/42)	100 (56/44)	70.70 ± 12.50	70.30 ± 10.25	Yixin decoction	*N*	① ③ ②	2 months	2.13 ± 0.76	1.15 ± 0.33	4.02 ± 1.04	1.55 ± 0.77	UA, HF, NFAMI, CD
Zhou et al. (22)	Post-PCI	55 (38/17)	50 (32/18)	64.7 ± 11.5	64.6 ± 11.2	Huazhuo Yixin Yin	*N*	① ②	3 months (6 months)	2.63 ± 0.15	1.02 ± 0.13	5.95 ± 0.42	1.98 ± 0.28	NFAMI, HF, AP
Tang and Qi (23)	Post-PCI	42 (25/17)	42 (24/18)	69 ± 8	69 ± 7	Tongmai Huayu decoction	*N*	①	6 months	2.92 ± 0.50	0.80 ± 0.24	5.4 ± 1.5	2.04 ± 0.49	AP, NFAMI, CD
Qin (24)	Post-PCI	38 (21/17)	38 (20/18)	65.1 ± 4.87	64.8 ± 4.6	Yiqi Huayu Tongluo recipe	*N*	② ① ⑤	4 weeks (6 months)	3.15 ± 0.51	1.84 ± 0.57	5.24 ± 0.71	3.24 ± 0.58	HF, NFS, NFAMI
Tang and Xiao (25)	ACS	75 (40/35)	75 (39/36)	51.47 ± 6.81	51.36 ± 6.45	Self-made prescription	*N*	② ① ⑦	15 days	4.33 ± 0.37	—	6.52 ± 1.21	2.30 ± 0.86	NFAMI, CD
Chenget al. (26)	Post-PCI	30 (20/10)	30 (22/8)	57.9 ± 5.2	58.1 ± 3.9	Anxin granules	*N*	① ⑦ ③ ②	6 months	3.73 ± 0.78	0.95 ± 0.25	6.91 ± 1.12	2.92 ± 1.04	HF, NFAMI, RV
Ma et al. (27)	UA	74 (31/43)	74 (34/40)	63.45 ± 8.24	63.71 ± 8.55	Yiqi Tongmai decoction	*N*	⑤ ① ③ ④ ②	8 weeks	2.83 ± 0.61	—	5.53 ± 1.39	2.28 ± 0.59	AP, NFAMI, HF
Zhang and Zhang (28)	AMI	38 (19/19)	38 (23/15)	59.0 ± 10.0	57.9 ± 10.2	Shenqi Fumai recipe	*N*	① ⑨⑦	4 weeks (6 months)	3.87 ± 0.08	1.87 ± 0.01	6.53 ± 0.29	2.15 ± 0.09	NFAMI, HF, CD
Li and Guo (29)	SA	40 (23/17)	40 (22/18)	62.84 ± 5.32	63.05 ± 5.14	Shenzhu Guanxin recipe	*N*	① ⑤	12 weeks (12 month)	3.24 ± 0.83	1.15 ± 0.29	5.21 ± 0.82	1.86 ± 0.62	NFAMI, UA, HF
Chen and Fan (30)	UA	30	30	—	—	Xintong recipe	*N*	⑤ ①	4 weeks (1 month)	2.85 ± 1.02	0.96 ± 0.34	4.97 ± 1.38	2.23 ± 1.20	RV, NFAMI
Lin and Yin (31)	UA	34 (21/13)	33 (18/15)	63	64	Wentong mixture	*N*	⑩ ① ②	6 months	4.18 ± 1.59	—	—	—	UA, NFAMI, CD
Zhao et al. (32)	ACS	34 (19/15)	34 (22/12)	59.60	57.91	Yiqi Huoxue Huayu recipe	*N*	⑤ ④ ⑩ ① ②	8 weeks	3.49 ± 1.17	2.18 ± 0.83	7.84 ± 1.26	2.69 ± 0.45	CD, NFAMI, AP, NFS
Kong et al. (33)	Post-PCI	39 (19/20)	40 (18/22)	59.3 ± 8.6	58.6 ± 10.4	Yugeng Tongyu decoction	*N*	④ ⑧	2 weeks (6 months)	4.51 ± 1.03	1.78 ± 0.79	6.43 ± 1.82	2.47 ± 0.60	CD, NFAMI, RV, NFS
Dai (34)	ACS	33 (20/13)	30 (19/11)	68.50	69.10	Shengxian decoction and Xiaoxianxiong decoction	*N*	⑩ ① ②	30 days (6 months)	1.67 ± 0.22	1.78 ± 0.64	4.07 ± 1.30	1.95 ± 0.77	CD, NFAMI, UA
Tan et al. (35)	Post-PCI	40 (31/9)	40 (30/10)	63.85 ± 10.85	62.65 ± 10.72	Yixin Tongmai recipe	*N*	① ② ③	4 weeks (1 year)	1.97 ± 0.79	1.11 ± 0.29	4.02 ± 0.91	1.72 ± 0.82	CD, AP, NFAMI
Li et al. (36)	ACS	30 (17/13)	30 (18/12)	61.8 ± 9.4	62.4 ± 9.15	Wenxin decoction	*N*	⑤ ① ⑦ ②	4 weeks (1 month)	4.94 ± 0.77	—	5.78 ± 1.14	1.58 ± 0.64	AP, CD, NFAMI
Sun et al. (37)	Borderline lesion CHD	43 (31/12)	45 (27/18)	65.3 ± 9.1	63.4 ± 9.5	Xiongshao capsule	*N*	⑤ ③ ⑩ ① ④ ②	12 months	3.95 ± 0.88	1.07 ± 0.29	5.04 ± 0.83	1.67 ± 1.05	AP, HF, NFAMI, NFS
Li and Long (38)	Borderline lesion CHD	80	76	—	—	Yiqi Wenyang Huoxue recipe	*N*	① ⑤ ③ ②	1 month (6 months)	3.01 ± 1.13	1.10 ± 0.78	4.89 ± 1.48	1.92 ± 0.83	NFAMI, AP, CD
Li et al. (39)	Borderline lesion CHD	120	120	61.06 ± 9.56	60.97 ± 10.04	Naoxintong capsule	*N*	⑤ ① ②	12 months	3.37 ± 0.72	—	—	—	CD, NFAMI, RV, AP
Zhao (40)	UA	48 (29/19)	30 (20/10)	—	—	Ningxin decoction	*N*	⑤	20 days (1 month)	2.47 ± 0.63	0.71 ± 0.21	5.19 ± 1.27	2.17 ± 1.20	MACEs
Lu et al. (41)	MI	2,429 (1991/438)	2,441 (2001/440)	58.97	58.83	Xuezhikang capsule	Placebo	① ③⑩ ④ ⑤	4.5 years	3.34 ± 0.65	1.19 ± 0.39	5.35 ± 0.67	1.85 ± 0.86	NFAMI, CD, RV

T, experimental group; C, control group; *N*, without intervention; AP, angina pectoris; AMI, acute myocardial infarction; NFAMI, non-fatal acute myocardial infarction; CD, cardiac death; RV, revascularization; NFS, non-fatal stroke; UA, unstable angina; SA, stable angina; ACS, acute coronary syndrome; HF, heart failure; post-PCI, post-percutaneous coronary intervention; MACEs, major adverse cardiovascular events; ① antiplatelet; ② statin; ③ β—blocker; ④ ACEI/ARB; ⑤ nitrate; ⑥ trimetazidine; ⑦ anticoagulant; ⑧ nicorandil; ⑨ urokinase; ⑩ calcium antagonist.

### 3.3 Study quality

All 23 included studies met the criterion of random allocation, 12 of the included 23 studies reported using a random number table, one applied random numbers generated by a computer, and 10 studies used randomization without providing methodological details. In addition, one study was conducted with blinding of participants and personnel, one study was conducted with blinding of outcome assessment, and four studies did not report the prespecified outcomes—the details are shown in Supplementary Material S2. Furthermore, the results of GRADE analysis demonstrated low confidence in the findings on CHM for lipid levels and CHM for MACEs stratified by lipid levels, the details are shown in Supplementary Material S2.

### 3.4 Efficacy of CHM on lipid profiles

#### 3.4.1 LDL-C levels

**Figure 2:**
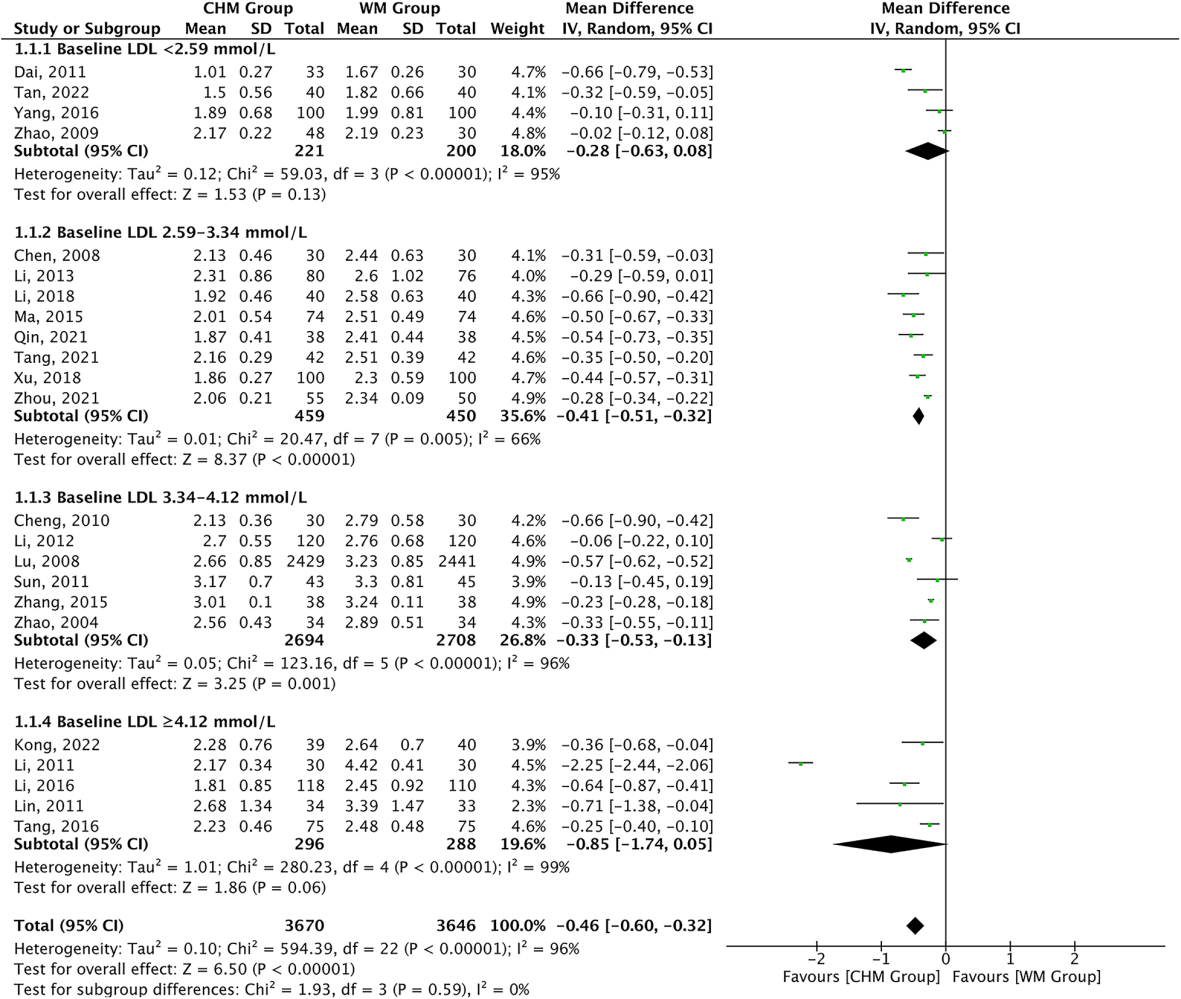
CHM for LDL-C level by baseline LDL-C level.

As presented in Table 1, 23 studies reported the LDL-C levels as the outcome measure. A significant difference was displayed in favor of CHM for lowering LDL-C levels compared with Western medicine treatment [MD = −0.46, 95% CI (−0.60 to −0.32), *P *< 0.00001, *I*^2 ^= 96%]. The reduction in LDL-C levels did not appear to be influenced by the baseline LDL-C level. The subgroup analysis according to baseline LDL-C level showed consistent LDL-C lowering effect of CHM across baseline levels of 2.59 mmol/L or greater (*P*_interaction_ = 0.59) (Figure 2). Furthermore, the efficiency of CHM for lowering LDL-C levels displayed significant heterogeneity in different subgroups. To find the potential source of heterogeneity, we conducted a meta-regression analysis and found that the heterogeneity might not be attributable to age, statin used, course of treatment, and type of disease (Supplementary Material S3). Deleting one trial at a time in the sensitivity analysis did not significantly reduce the heterogeneity except the trial of Dai (34), which slightly reduced the heterogeneity (*I*^2 ^= 74.6%, *P *= 0.008) (Supplementary Material S4). It should be noted that the trial of Dai was different from that of others, with an LDL-C below 1.8 mmol/L.

#### 3.4.2 TG levels

**Figure 3:**
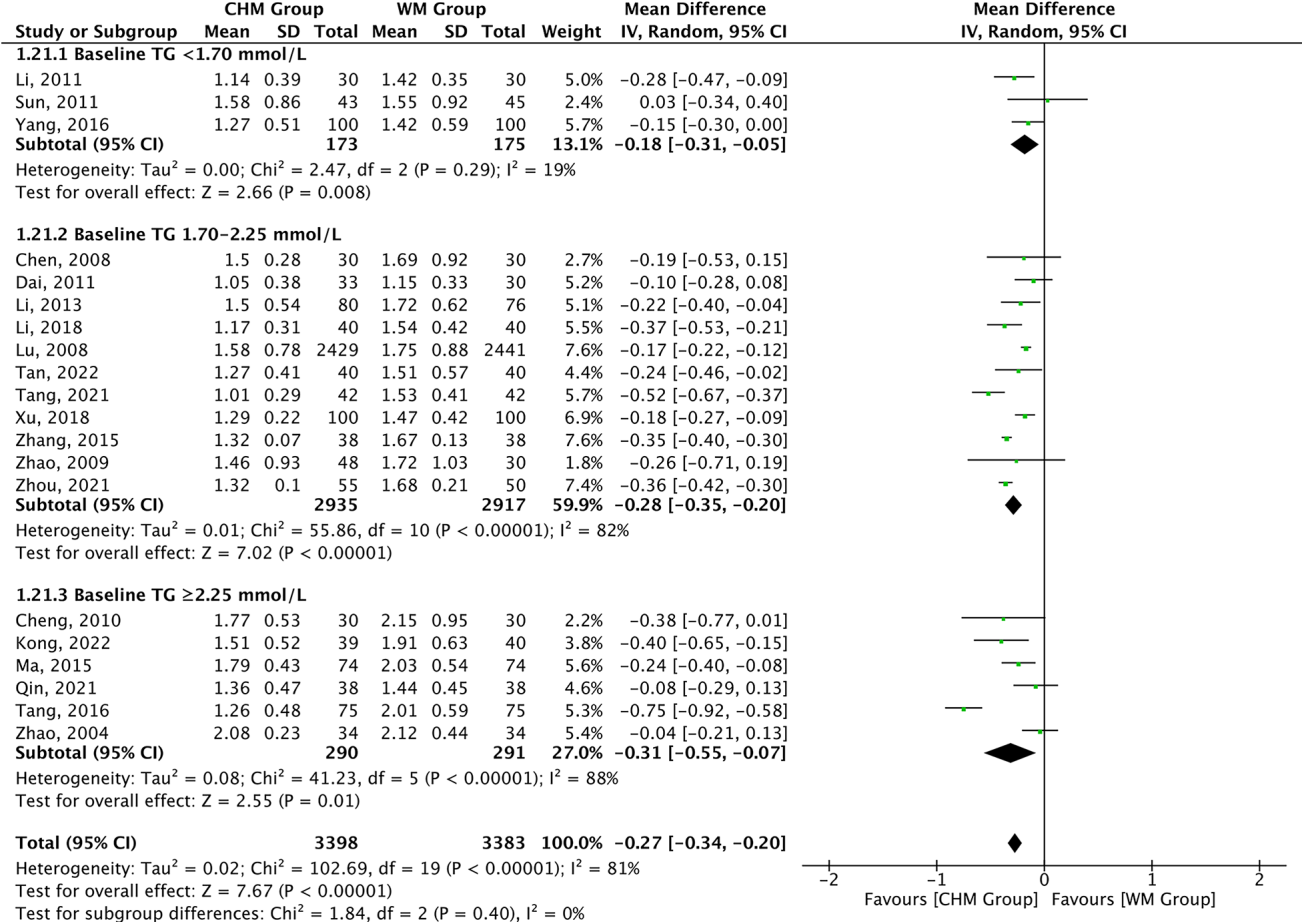
CHM for TG level by baseline TG level.

TG levels were reported as the outcome measures in 20 studies. A significant difference was found in favor of CHM for lowering TG levels compared with Western medicine treatment [MD = −0.27, 95% CI (−0.34 to −0.20), *P *< 0.00001, *I*^2 ^= 81%]. Subgroup analysis according to baseline TG level showed consistent TG lowering effect of CHM across the baseline subgroup (*P*_interaction _= 0.40) (Figure 3). The efficiency of CHM for regulating TG levels displayed significant heterogeneity in different subgroups. The meta-regression analysis showed that the heterogeneity might be attributable to the course of treatment: <6 months [16 trials, MD = −0.27, 95% CI (−0.34 to −0.19)] vs. 6–12 months [2 trials, MD = −0.50, 95% CI (−0.64 to −0.36)] vs. ≥12 months [2 trials, MD = −0.16, 95% CI (−0.25 to −0.07)], *P *= 0.0003 for interaction, and might not be attributable to age, statin used, and type of disease (Supplementary Material S3).

#### 3.4.3 TC levels

**Figure 4:**
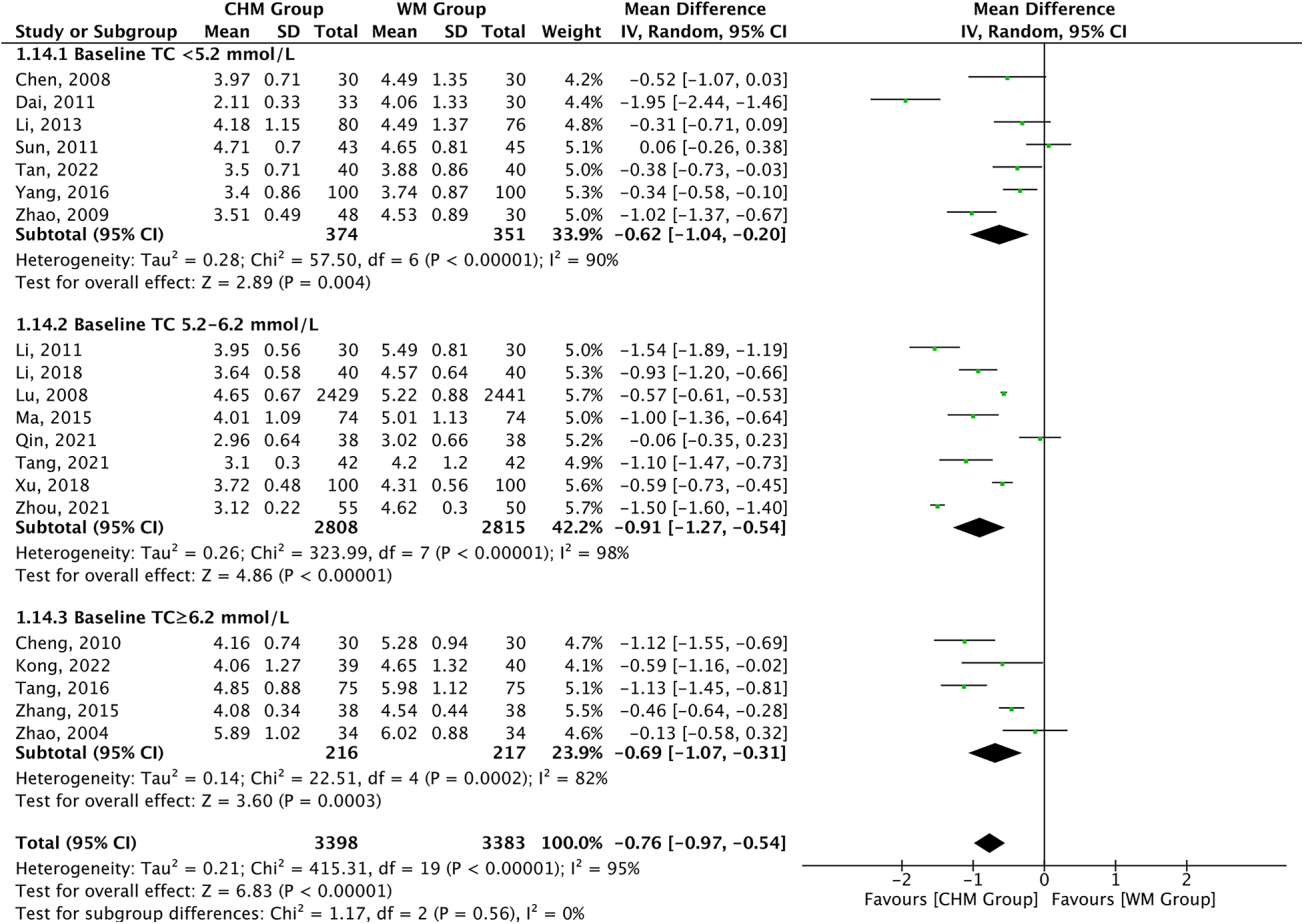
CHM for TC level by baseline TC level.

TC levels were reported as the outcome measures in 20 studies. A significant difference was found in favor of CHM for lowering TC levels compared with Western medicine therapy [MD = −0.76, 95% CI (−0.97 to −0.54), *P *< 0.00001, *I*^2 ^= 95%]. The subgroup analysis according to baseline TC level showed consistent TC lowering effect of CHM across the baseline subgroup (*P*_interaction _= 0.56) (Figure 4). The efficiency of CHM for regulating TC levels displayed significant heterogeneity in different subgroups, which might be a result of the treatment duration as shown in the meta-regression analysis: <6 months [16 trials, MD = −0.78, 95% CI (−1.06 to −0.49)] vs. 6–12 months [2 trials, MD = −1.11, 95% CI (−1.39 to −0.83)] vs. ≥12 months [2 trials, MD = −0.28, 95% CI (−0.89 to 0.34)], *P *= 0.03 for interaction, and might not be attributable to age, statin used, and type of disease (Supplementary Material S3).

#### 3.4.4 HDL-C levels

**Figure 5:**
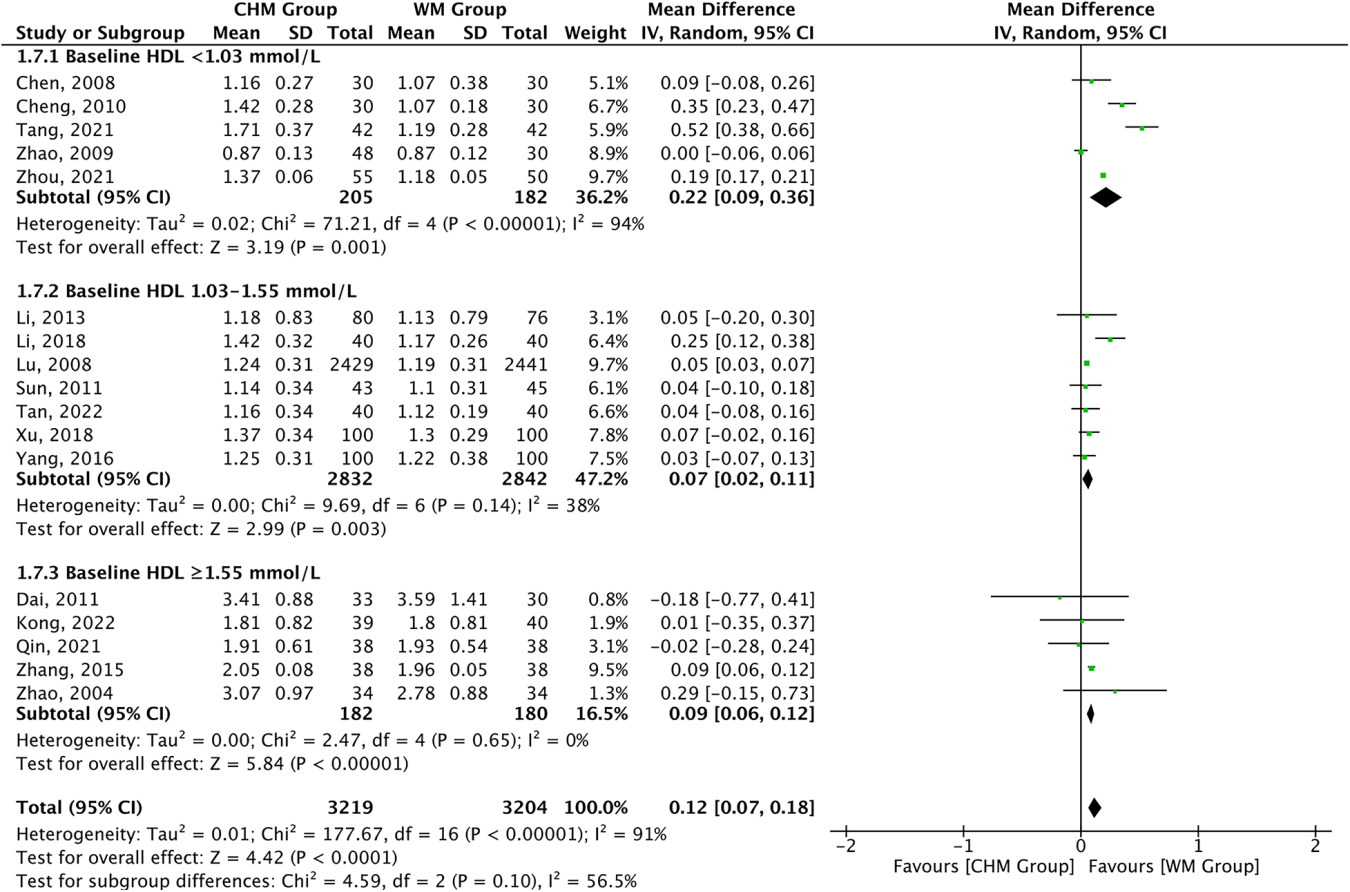
CHM for HDL-C level by baseline HDL-C level.

In total, 17 studies reported the HDL-C levels as the outcome measures. A significant difference was found in favor of CHM for increasing HDL-C levels compared with Western medicine treatment [MD = 0.12, 95% CI (0.07–0.18), *P* < 0.0001, *I*^2 ^= 91%]. The subgroup analysis according to baseline HDL-C level showed consistent HDL-C increasing effect of CHM across baseline subgroup (*P*_interaction_*_ _*= 0.10) (Figure 5). The efficiency of CHM for regulating HDL-C levels displayed significant heterogeneity in different subgroups. The meta-regression analysis displayed that the heterogeneity present in the trials appears to be attributable to the course of treatment: <6 months [13 trials, MD = 0.09, 95% CI (0.03–0.15)] vs. 6–12 months [2 trials, MD = 0.43, 95% CI (0.26–0.60)] vs. ≥12 months [2 trials, MD = 0.05, 95% CI (0.03–0.07)], *P* < 0.0001 for interaction, and might not be attributable to age, statin used, and type of disease (Supplementary Material S3).

### 3.5 Efficacy of CHM on MACEs

#### 3.5.1 Cardiovascular events by baseline LDL-C level

**Figure 6:**
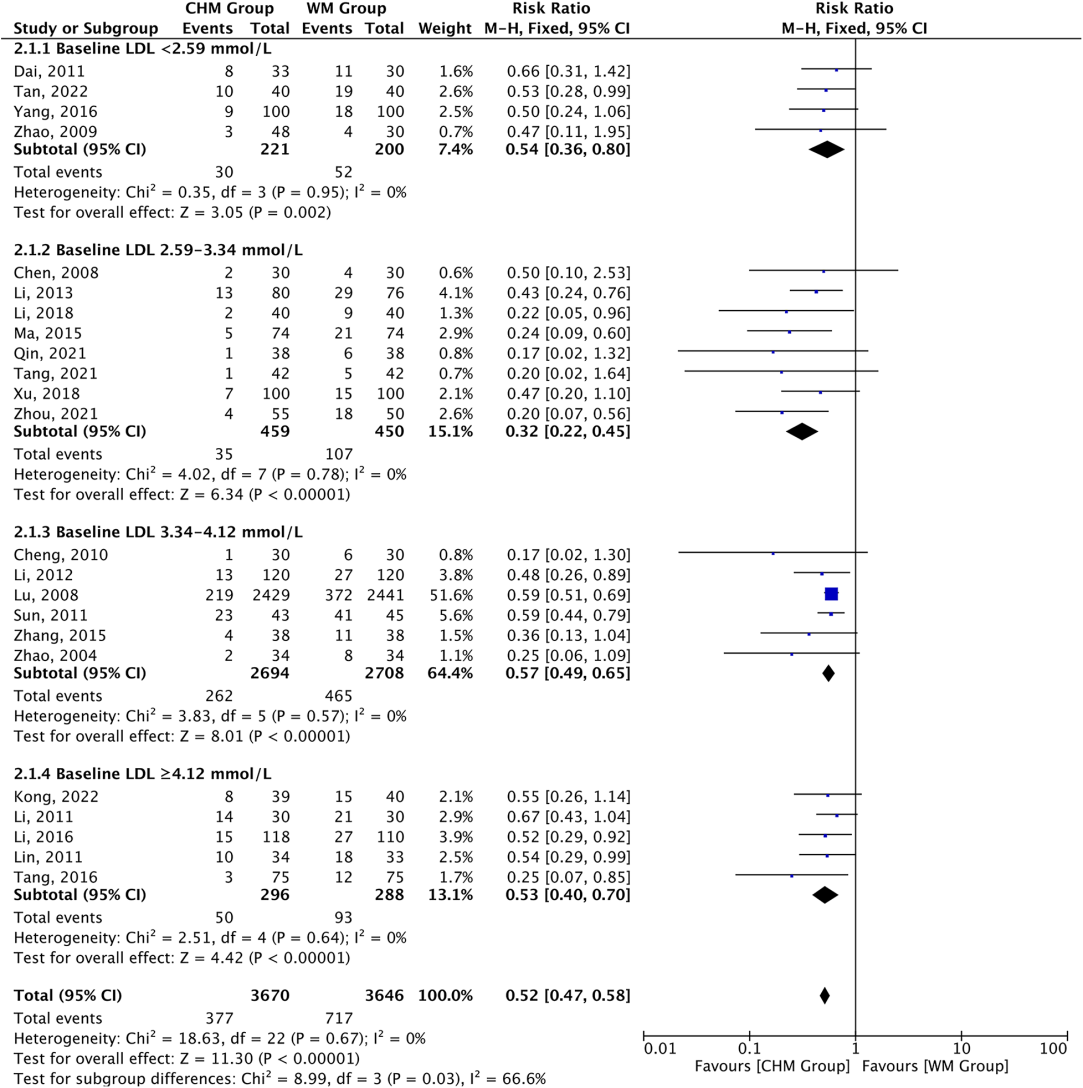
CHM for MACEs stratified by baseline LDL-C level.

Overall, 377 of the 3,670 patients receiving CHM therapy vs. 717 of the 3,646 receiving Western medicine therapy experienced cardiovascular events during follow-up, the composite endpoint MACEs definitions across studies are provided in Table 1. A 48% risk reduction for MACEs was observed in the CHM group vs. the control group [RR 0.52, 95%CI (0.47–0.58), *P *< 0.00001, *I*^2 ^= 0%] and the risk reduction varied according to the baseline LDL-C level (*P*_interaction _= 0.03). Furthermore, the risk reduction in MACEs appeared to be influenced by the baseline LDL-C level and not by the magnitude of LDL-C reduction. The risk of MACEs was related to CHM therapy in all subgroups of baseline LDL-C level, ranging from baseline levels less than 2.59 mmol/L [RR 0.54, 95% CI (0.36–0.80), *P *= 0.002, *I*^2 ^= 0%] to baseline levels of 4.12 mmol/L or greater [RR 0.53, 95% CI (0.40–0.70), *P *< 0.00001, *I*^2 ^= 0%], with a significant *P*-value (*P *= 0.03) for interaction in the subgroup (Figure 6). The subgroup with baseline LDL-C levels of 2.59–3.34 mmol/L showed the highest reductions [RR 0.32, 95% CI (0.22–0.45), *P *< 0.00001, *I*^2 ^= 0%]. However, risk of MACEs was associated with CHM therapy in all subgroups with magnitude of LDL-C reduction, but there was a non-significant *P*-value (*P *= 0.34) for interaction (Supplementary Material S5). The above results remained unchanged after analysis using the random effect model (Supplementary Material S6).

#### 3.5.2 Cardiovascular events by baseline TG level

**Figure 7:**
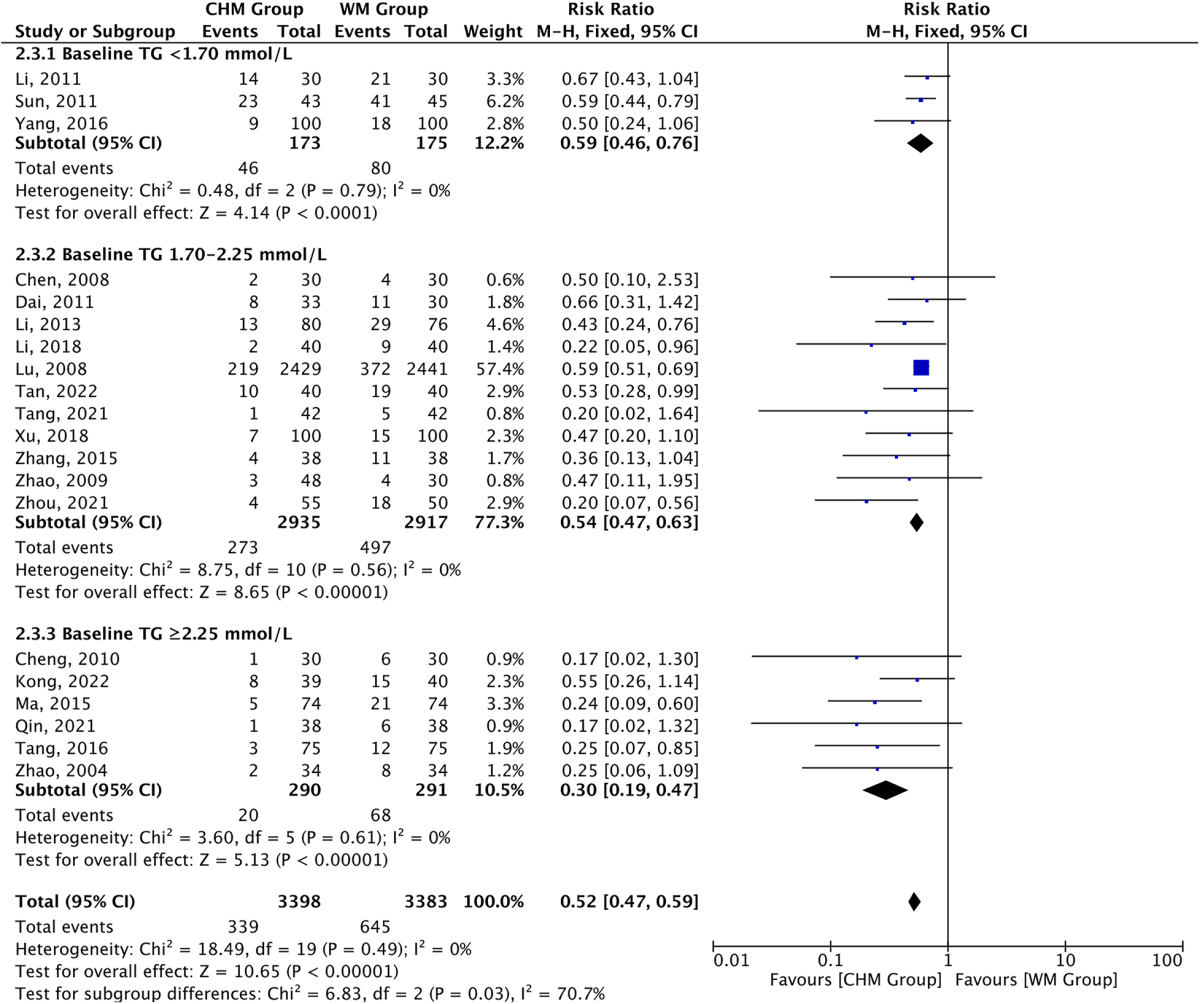
CHM for MACEs by baseline TG level.

Cardiovascular events occurred in 339 of the 3,398 patients receiving CHM therapy vs. 645 of the 3,383 receiving Western medicine during follow-up. A 48% risk reduction for MACEs was observed in the CHM group vs. the control group [RR 0.52, 95% CI (0.47–0.59), *P *< 0.00001, *I*^2 ^= 0%] and the risk reduction varied according to baseline TG level (*P*_interaction_ = 0.03). Furthermore, the risk reduction in MACEs appeared to be influenced by both the baseline TG level and the magnitude of TG reduction. The risk of MACEs was related to CHM therapy in all subgroups of baseline TG levels, ranging from baseline levels less than 1.70 mmol/L [RR 0.59, 95% CI (0.46–0.76), *P *< 0.0001, *I*^2 ^= 0%] to baseline levels of 2.25 mmol/L or greater [RR 0.30, 95% CI (0.19–0.47), *P *< 0.00001, *I*^2 ^= 0%], with a significant *P*-value (*P *= 0.03) for interaction in the different subgroups. In addition, higher baseline TG levels were associated with progressively greater risk reduction in MACEs (Figure 7). A further analysis revealed that risk of MACEs was associated with CHM therapy in all subgroups with magnitude of TG reduction, with a significant *P*-value (*P *= 0.02) for interaction. Furthermore, the greater the magnitude of TG reduction, the greater the reduction of risk for MACEs (Supplementary Material S5). The above results remained unchanged after analysis using the random effect model (Supplementary Material S6).

#### 3.5.3 Cardiovascular events by baseline TC level

A total of 339 of the 3,398 patients receiving CHM therapy vs. 645 of the 3,383 receiving Western medicine experienced cardiovascular events during follow-up. A 48% risk reduction for MACEs was observed in the CHM group vs. the control group [RR 0.52, 95% CI (0.47–0.59), *P *< 0.00001, *I*^2 ^= 0%] and the risk reduction varied according to baseline TC levels (*P*_interaction_ = 0.24). Furthermore, the risk reduction in MACEs appeared to be influenced by the magnitude of TC reduction and not by the baseline TC level, and a greater magnitude in TC reduction was associated with progressively greater risk reduction in MACEs (Supplementary Material S5).

#### 3.5.4 Cardiovascular events by baseline HDL-C level

Altogether 317 of the 3,219 patients receiving CHM therapy vs. 591 of the 3,204 receiving Western medicine were reported to experience cardiovascular events during follow-up. A 47% risk reduction for MACEs was observed in the CHM group vs. the control group [RR 0.53, 95%CI (0.47–0.61), *P *< 0.00001, *I*^2 ^= 0%] and the risk reduction varied according to baseline HDL-C level (*P*_interaction_ = 0.05). Furthermore, the risk reduction in MACEs did not appear to be influenced by the baseline HDL-C level and magnitude of HDL-C reduction (Supplementary Material S5).

### 3.6 Additional analysis

We also analyzed each component of the MACEs and found that additional CHM treatment was associated with a significant risk reduction in cardiovascular mortality, myocardial infarction, revascularization, angina pectoris, and heart failure, but not with non-fatal stroke (*P *= 0.05) (Supplementary Material S7). As a meta-regression analysis, the reduction in risk of total MACEs treated by CHM therapy was associated with course of treatment, *P *= 0.03 for interaction, and might not be attributable to age, statin used, and type of disease (Supplementary Material S8).

Funnel plots and Egger's test were asymmetric for the MD of lipid levels and RR for MACEs, presenting potential publication bias (Supplementary Material S9). A further sensitivity analysis was conducted to show the influence of each trial by deleting each in turn. Deleting each trial successively did not lead to significant deviation from the original overall estimate, indicating that the association of CHM for reducing the risk of MACEs is robust (Supplementary Material S10).

The adverse events during the treatment were reported by three studies. However, there is no significant difference of adverse event rate between the CHM and the Western medicine groups [OR 1.10, 95% CI (0.64–1.89), *P *= 0.72, *I*^2 ^= 7%] (Supplementary Material S11).

Given the formulae were different due to the type and severity of patients, the frequency statistics of CHM were analyzed to determine the commonly used drugs among different trials. Sichuan lovage rhizome (Chuanxiong, Rhizoma Ligustici Chuanxiong), Danshen root (Danshen, Radix Salviae Miltiorrhizae), Snakegourd fruit (Gualou, Fructus Trichosanthis), Astragalus (Huangqi, Radix Astragali Membranacei), and Peony root (Chishao, Radix Paeoniae Rubra) were the most commonly used in the treatment of CHD (Supplementary Material S12).

## 4 Discussion

### 4.1 Summary of main results

In this study, a systematic review and meta-analysis of 23 randomized controlled trials (RCTs) of CHM for regulating lipid levels and lowering cardiovascular events among patients with CHD were conducted, covering 7,316 participants, and 377 cardiovascular events occurred in the 3,670 patients in the CHM group, while 717 events were reported in the 3,646 patients in the Western medicine alone group. First, CHM therapy compared with Western medicine alone was associated with significantly improved lipid profiles.

Second, compared with Western medicine alone, the addition of CHM was associated with a lower risk of cardiovascular events, and the strength of the association varied according to baseline LDL-C and TG levels, and the magnitude in TG and TC reduction. Greater risk reduction in MACEs was observed in patients with baseline LDL-C levels of 2.59–3.34 mmol/L and higher baseline TG level.

Third, CHM therapy was also associated with greater reduction in risk of cardiovascular mortality, myocardial infarction, angina pectoris, revascularization, and heart failure. By contrast, the reduction in non-fatal strokes did not appear to be related to CHM therapy. Considering the probability that the relatively short duration of follow-up is implicated in the lack of MACEs, it could be hypothesized that the course of treatment of CHM and duration of follow-up are too short to fully demonstrate the clinical benefits. It may also be due to the small number of participants. Therefore, further observation is needed for the long-term prognosis.

### 4.2 Possible explanations of the findings

The development of dyslipidemia, characterized by plasma hypertriglyceridemia, remnant lipoprotein of cholesterol levels (especially elevated LDL-C levels), and decreased HDL-C levels, is a critical driver for atherosclerotic plaques, which further exacerbate risk of MACEs. In a meta-regression analysis, TG lowering is associated with cardiovascular risk reduction, even after adjusting for LDL-C lowering (8). Recent genetic data highlight that interventions of lowering TG levels are also associated with cardiovascular benefits (42). Specifically, it is found that a decrease in 40 mg/dl of LDL-C should lower by approximately 20% the risk of cardiovascular events, but a similar 40 mg/dl reduction in TG levels could generate a 4%–5% reduction of cardiovascular events. Furthermore, the association between TG and MACEs is well demonstrated from epidemiological studies (43, 44) and Mendelian randomization studies (45, 46). Therefore, it will be difficult to achieve a cardiovascular benefit with a purely LDL-C or TG lowering.

Elevated levels of LDL-C and TG are associated with MACEs, all clinicians attest to the fact that intensive LDL-C or TG lowering in cardiovascular disease prevention, although helpful, is difficult to achieve in practice (47). Although the omega-3-fatty acids eicosapentaenoic acid (EPA) and docosahexaenoic acid (DHA) could lower TG level by 10%–50%, LDL-C may increase with EPA + DHA when TG levels are high (>5,000 mg/dl). In addition, the STRENGTH trial showed that high dose EPA + DHA did not reduce risk of cardiovascular events (48). Therapy purely with fibrates was found to reduce risk of cardiovascular events in patients with high TG and low HDL-C levels. It is currently uncertain whether reduction of cardiovascular events is associated with the addition of fibrates to statin therapy, which may increase the risk of muscle symptoms (49).

Different from Western medicine, characterized by multi-components, multi-targets, and multi-pathways, CHM plays an important role in antiatherosclerosis effects, cardioprotective function, and regulation of lipid levels, which is widely used for CHD in China (50–52). The Huazhuo Yixin Yin is a Chinese medicine decoction, which has been used for treatment of CHD over several years. Zhou et al. (22) reported that Huazhuo Yixin Yin could reduce the risk of MACEs, which was partly the result of its lipid metabolism regulation. Lu et al. (41) administered long-term therapy with Xuezhikang to patients with previous myocardial infarction and found that the incidence of MACEs was significantly decreased and the lipoprotein regulation was improved, and also safe and well tolerated. In these trials, additional CHM administration provided additional reduction in MACEs. Yuan et al. (53) found that the Chuanxiong–Chishao herb pair could improve cardiac function and reduce myocardial fibrosis area by regulating the expression of circRNAs and IncRNAs and its intervention mechanism in CHD may be related to the regulation of lipid metabolism, lipid transport, inflammation, and angiogenesis. Tetramethylpyrazine is an active alkaloid in Ligusticum chuanxiong Hort, which can exert protective effects on myocardial ischemia injury, including scavenging oxygen free radicals (OFRs) to inhibit lipid peroxidation, inhibiting apoptosis and anti-inflammatory activity (54). Ma et al. (55) reported that *Salvia miltiorrhiza* and Tanshinone IIA could attenuate the buildup of plaque and the accumulation of lipid in ApoE^−/−^ mice and its mechanism may be related to reducing vascular endothelial inflammation and preventing plaque formation via COX-2/TNF-α/NF-κB signaling pathway. Li et al. (56) summarized that the lipid-regulating effect of CHM in CVD may be related to inhibiting intestinal absorption of lipids, reducing endogenous cholesterol synthesis, regulating transcription factors related to lipid metabolism, regulating cholesterol transport, and promoting the excretion of cholesterol in the liver. These findings suggest that CHM may be used as a complementary approach for patients with CHD. Nevertheless, more rigorously designed RCTs are needed to support these findings.

### 4.3 Limitations

This study has several limitations. First, the analyses were at the trial level, only the association between risk of cardiovascular events and lipid-lowering treatment by CHM within a trial was considered. Considering the differences in patients, comorbidities (e.g., diabetes), and treatment formulae, subgroup analyses might provide additional explanations on sources of heterogeneity. Second, the lipid levels at the end of the treatment period were used rather than at the end of the follow-up period, which may have overlooked the association of the magnitude of lipid lowering during the follow-up. Third, the formulae were different due to the type and severity of illness of the patients; most prescriptions were modified depending on the individual physique and clinical presentations. Fourth, the included trials lacked strict trial design and adequate sample size estimation. The blinding of participants, personnel, and outcome assessment was performed in 2 of 23 studies.

### 4.4 Implications for clinical practice and research

Given the higher reduction in lipid levels and risk of cardiovascular events, CHM therapy could be considered in patients with high baseline LDL-C and TG levels as it also would offer clinical benefits from lipid lowering. In addition, if additional lipid-lowering therapies or additional treatment to reduce adverse effects are considered for patients treated with Western medicine , CHM therapy to reduce cardiovascular risk is recommended. Although the possible mechanism of CHM in CHD treatment has been discussed, further exploration of the active ingredients and cardioprotective mechanisms of CHM in CHD treatment is of great significance. Therefore, because of the residual cardiovascular risk of Western medicine in the treatment of CHD, more RCTs using multiple centers, larger samples, and strict methodologies are necessary to explore the effectiveness and cardioprotective mechanisms of CHM therapy for CHD.

## 5 Conclusion

CHM compared with Western medicine alone was associated with lower risk of cardiovascular events and improvement of lipid profile. Risk reduction for cardiovascular events was associated with baseline LDL-C and TG levels, and the magnitude in TG and TC reduction. Greater risk reduction in MACEs was observed in patients with baseline LDL-C levels of 2.59–3.34 mmol/L and higher baseline TG levels.

## Data Availability

The original contributions presented in the study are included in the article/**Supplementary Material**, further inquiries can be directed to the corresponding authors.
