# Supplementary material for: Traditional Chinese medicine lowering lipid levels and cardiovascular events across baseline lipid levels among coronary heart disease: a meta-analysis of randomized controlled trials
Source: Front Cardiovasc Med. 2024 Jul 11;11:1407536. doi: 10.3389/fcvm.2024.1407536 (PMC11269158; doi:10.3389/fcvm.2024.1407536)
Supplement: Supplementary file 1 [file Table1.docx]

# Supplementary material S1. Search strategy of six database

## English search strategy

## Web of Science

#1: ("Coronary Disease" OR "Coronary Diseases" OR "Disease, Coronary" OR "Diseases, Coronary" OR "Coronary Heart Disease" OR "Coronary Heart Diseases" OR "Disease, Coronary Heart" OR "Diseases, Coronary Heart" OR "Heart Disease, Coronary" OR "Heart Diseases, Coronary")

#2:("coronary artery disease" OR "Artery Disease, Coronary" OR "Artery Diseases, Coronary" OR "Coronary Artery Diseases" OR "Left Main Coronary Artery Disease" OR "Left Main Disease" OR "Left Main Diseases" OR "Left Main Coronary Disease" OR "Coronary Arteriosclerosis" OR "Arterioscleroses, Coronary" OR "Coronary Arterioscleroses" OR "Atherosclerosis, Coronary" OR "Atheroscleroses, Coronary" OR "Coronary Atheroscleroses" OR "Coronary Atherosclerosis" OR "Arteriosclerosis, Coronary")

#3:("Myocardial Infarction" OR "Infarction, Myocardial" OR "Infarctions, Myocardial" OR "Myocardial Infarctions" OR "Cardiovascular Stroke" OR "Cardiovascular Strokes" OR "Stroke, Cardiovascular" OR "Strokes, Cardiovascular" OR "Myocardial Infarct" OR "Infarct, Myocardial" OR "Infarcts, Myocardial" OR "Myocardial Infarcts" OR "Heart Attack" OR "Heart Attacks" OR "Angina pectoris" OR "Angina cordis" OR "Stenocardia")

#4:("Acute Coronary Syndrome" OR "Acute Coronary Syndromes" OR "Coronary Syndrome, Acute" OR "Coronary Syndromes, Acute" OR "Syndrome, Acute Coronary" OR "Syndromes, Acute Coronary")

#5: (((#1) OR #2) OR #3) OR #4

#6: ("Medicine, Chinese Traditional" OR "Traditional Chinese Medicine" OR "Traditional Medicine, Chinese" OR "Zhong Yi Xue" OR "Chinese Traditional Medicine" OR "Chinese Medicine, Traditional")

#7: ("Herbal medicine" OR "Medicine, Herbal" OR "herb" OR "Chinese herbal medicine")

#8: ((#6) OR #7)

#9: ("blood fat" OR "blood lipid" OR "lipid metabolism" OR "lipid" OR "LDL-C" OR "low-density lipoprotein" OR "HDL-C" OR "high-density lipoprotein" OR "lipoprotein" OR "cholesterol" OR "triglyceride")

#10 ("Randomized controlled trail" OR "Randomized" OR "Placebo" OR "clinical study" OR "clinical observation")

#11: ((((#5) AND #8) AND #9) AND #10)

## Cochrane Library

#1: (Coronary Disease):ti,ab,kw OR (Coronary Diseases):ti,ab,kw OR (Disease, Coronary):ti,ab,kw OR (Diseases, Coronary):ti,ab,kw OR (Coronary Heart Disease):ti,ab,kw OR (Coronary Heart Diseases):ti,ab,kw OR (Disease, Coronary Heart):ti,ab,kw OR (Diseases, Coronary Heart):ti,ab,kw OR (Heart Disease, Coronary):ti,ab,kw OR (Heart Diseases, Coronary):ti,ab,kw

#2: (coronary artery disease):ti,ab,kw OR (Artery Disease, Coronary):ti,ab,kw OR (Artery Diseases, Coronary):ti,ab,kw OR (Coronary Artery Diseases):ti,ab,kw OR (Left Main Coronary Artery Disease):ti,ab,kw OR (Left Main Disease):ti,ab,kw OR (Left Main Diseases):ti,ab,kw OR (Left Main Coronary Disease):ti,ab,kw OR (Coronary Arteriosclerosis):ti,ab,kw OR (Arterioscleroses, Coronary):ti,ab,kw OR (Coronary Arterioscleroses):ti,ab,kw OR (Atherosclerosis, Coronary):ti,ab,kw OR (Atheroscleroses, Coronary):ti,ab,kw OR (Coronary Atheroscleroses):ti,ab,kw OR (Coronary Atherosclerosis):ti,ab,kw OR (Arteriosclerosis, Coronary):ti,ab,kw

#3: (Myocardial Infarction):ti,ab,kw OR (Infarction, Myocardial):ti,ab,kw OR (Infarctions, Myocardial):ti,ab,kw OR (Myocardial Infarctions):ti,ab,kw OR (Cardiovascular Stroke):ti,ab,kw OR (Cardiovascular Strokes):ti,ab,kw OR (Stroke, Cardiovascular):ti,ab,kw OR (Strokes, Cardiovascular):ti,ab,kw OR (Myocardial Infarct):ti,ab,kw OR (Infarct, Myocardial):ti,ab,kw OR (Infarcts, Myocardial):ti,ab,kw OR (Myocardial Infarcts):ti,ab,kw OR (Heart Attack):ti,ab,kw OR (Heart Attacks):ti,ab,kw OR (Angina pectoris):ti,ab,kw OR (Angina cordis):ti,ab,kw OR (Stenocardia):ti,ab,kw

#4: (Acute Coronary Syndrome):ti,ab,kw OR (Acute Coronary Syndromes):ti,ab,kw OR (Coronary Syndrome, Acute):ti,ab,kw OR (Coronary Syndromes, Acute):ti,ab,kw OR (Syndrome, Acute Coronary):ti,ab,kw OR (Syndromes, Acute Coronary):ti,ab,kw

#5: (((#1) OR #2) OR #3) OR #4

#6: (Medicine, Chinese Traditional):ti,ab,kw OR (Traditional Chinese Medicine):ti,ab,kw OR (Traditional Medicine, Chinese):ti,ab,kw OR (Zhong Yi Xue):ti,ab,kw OR (Chinese Traditional Medicine):ti,ab,kw OR (Chinese Medicine, Traditional):ti,ab,kw

#7: (Herbal medicine):ti,ab,kw OR (Medicine, Herbal):ti,ab,kw OR (herb):ti,ab,kw OR (Chinese herbal medicine):ti,ab,kw

#8: ((#6) OR #7)

#9: (blood fat):ti,ab,kw OR (blood lipid):ti,ab,kw OR (lipid metabolism):ti,ab,kw OR (lipid):ti,ab,kw OR (LDL-C):ti,ab,kw OR (low-density lipoprotein):ti,ab,kw OR (HDL-C):ti,ab,kw OR (high-density lipoprotein):ti,ab,kw OR (lipoprotein):ti,ab,kw OR (cholesterol):ti,ab,kw OR (triglyceride):ti,ab,kw

#10 (Randomized controlled trail):ti,ab,kw OR (Randomized):ti,ab,kw OR (Placebo):ti,ab,kw OR (clinical study):ti,ab,kw OR (clinical observation):ti,ab,kw

#11: ((((#5) AND #8) AND #9) AND #10)

## PubMed

#1: (((((((((Coronary Disease[Title/Abstract]) OR (Coronary Diseases[Title/Abstract])) OR (Disease, Coronary[Title/Abstract])) OR (Diseases, Coronary[Title/Abstract])) OR (Coronary Heart Disease[Title/Abstract])) OR (Coronary Heart Diseases[Title/Abstract])) OR (Disease, Coronary Heart[Title/Abstract])) OR (Diseases, Coronary Heart[Title/Abstract])) OR (Heart Disease, Coronary[Title/Abstract])) OR (Heart Diseases, Coronary[Title/Abstract])

#2: (((((((((((((((coronary artery disease[Title/Abstract]) OR (Artery Disease, Coronary[Title/Abstract])) OR (Artery Diseases, Coronary[Title/Abstract])) OR (Coronary Artery Diseases[Title/Abstract])) OR (Left Main Coronary Artery Disease[Title/Abstract])) OR (Left Main Disease[Title/Abstract])) OR (Left Main Diseases[Title/Abstract])) OR (Left Main Coronary Disease[Title/Abstract])) OR (Coronary Arteriosclerosis[Title/Abstract])) OR (Arterioscleroses, Coronary[Title/Abstract])) OR (Coronary Arterioscleroses[Title/Abstract])) OR (Atherosclerosis, Coronary[Title/Abstract])) OR (Atheroscleroses, Coronary[Title/Abstract])) OR (Coronary Atheroscleroses[Title/Abstract])) OR (Coronary Atherosclerosis[Title/Abstract])) OR (Arteriosclerosis, Coronary[Title/Abstract])

#3: ((((((((((((((((Myocardial Infarction[Title/Abstract]) OR (Infarction, Myocardial[Title/Abstract])) OR (Infarctions, Myocardial[Title/Abstract])) OR (Myocardial Infarctions[Title/Abstract])) OR (Cardiovascular Stroke[Title/Abstract])) OR (Cardiovascular Strokes[Title/Abstract])) OR (Stroke, Cardiovascular[Title/Abstract])) OR (Strokes, Cardiovascular[Title/Abstract])) OR (Myocardial Infarct[Title/Abstract])) OR (Infarct, Myocardial[Title/Abstract])) OR (Infarcts, Myocardial[Title/Abstract])) OR (Myocardial Infarcts[Title/Abstract])) OR (Heart Attack[Title/Abstract])) OR (Heart Attacks[Title/Abstract])) OR (Angina pectoris[Title/Abstract])) OR (Angina cordis[Title/Abstract])) OR (Stenocardia[Title/Abstract])

#4: (((((Acute Coronary Syndrome[Title/Abstract]) OR (Acute Coronary Syndromes[Title/Abstract])) OR (Coronary Syndrome, Acute[Title/Abstract])) OR (Coronary Syndromes, Acute[Title/Abstract])) OR (Syndrome, Acute Coronary[Title/Abstract])) OR (Syndromes, Acute Coronary[Title/Abstract])

#5: (((#1) OR #2) OR #3) OR #4

#6: (((((Medicine, Chinese Traditional[Title/Abstract]) OR (Traditional Chinese Medicine[Title/Abstract])) OR (Traditional Medicine, Chinese[Title/Abstract])) OR (Zhong Yi Xue[Title/Abstract])) OR (Chinese Traditional Medicine[Title/Abstract])) OR (Chinese Medicine, Traditional[Title/Abstract])

#7: (((Herbal medicine [Title/Abstract]) OR (Medicine, Herbal [Title/Abstract])) OR (herb [Title/Abstract])) OR (Chinese herbal medicine [Title/Abstract])

#8: ((#6) OR #7)

#9: ((((((((((blood fat[Title/Abstract]) OR (blood lipid[Title/Abstract])) OR (lipid metabolism[Title/Abstract])) OR (lipid[Title/Abstract])) OR (LDL-C[Title/Abstract])) OR (low-density lipoprotein[Title/Abstract])) OR (HDL-C[Title/Abstract])) OR (high-density lipoprotein[Title/Abstract])) OR (lipoprotein[Title/Abstract])) OR (cholesterol[Title/Abstract])) OR (triglyceride[Title/Abstract])

#10: ((((Randomized controlled trail [Title/Abstract]) OR (Randomized [Title/Abstract])) OR (Placebo [Title/Abstract])) OR (clinical study [Title/Abstract])) OR (clinical observation [Title/Abstract])

#11: ((((#5) AND #8) AND #9) AND #10

## Chinese search strategy

## CNKI

SU=('冠心病'+'冠状动脉粥样硬化心脏病'+'冠状动脉粥样硬化性心脏病'+'急性冠脉综合征'+'急性冠脉综合症'+'急性冠状动脉综合征'+'急性冠状动脉综合症'+'心肌梗死'+'心梗'+'心肌梗塞'+'心绞痛')*('中医'+'中药'+'中医药'+'草药'+'中草药'+'方剂'+'药方'+'颗粒'+'中成药')*('血脂'+'脂质代谢'+'脂质'+'低密度脂蛋白'+'脂蛋白'+'LDL-C'+'胆固醇'+'甘油三酯'+'高密度脂蛋白'+'HDL-C')*('随机对照试验'+'随机对照研究'+'RCT'+'随机'+'临床研究'

## WangFang Database

主题：("冠心病" or "冠状动脉粥样硬化心脏病" or "冠状动脉粥样硬化性心脏病" or "急性冠脉综合征" or "急性冠脉综合症" or "急性冠状动脉综合征" or "急性冠状动脉综合症" or "心肌梗死" or "心梗" or "心肌梗塞" or "心绞痛") and ("中医" or "中药" or "中医药" or "草药" or "中草药" or "方剂" or "药方" or "颗粒" or "中成药") and ("血脂" or "脂质代谢" or "脂质" or "低密度脂蛋白" or "脂蛋白" or "LDL-C" or "胆固醇" or "甘油三酯" or "高密度脂蛋白" or "HDL-C") and ("随机对照试验" or "随机对照研究" or "RCT" or "随机" or "临床研究")

## VIP Information Database

M=(冠心病 OR 冠状动脉粥样硬化心脏病 OR 冠状动脉粥样硬化性心脏病 OR 急性冠脉综合征 OR 急性冠脉综合症 OR 急性冠状动脉综合征 OR 急性冠状动脉综合症 OR 心肌梗死 OR 心梗 OR 心肌梗塞 OR 心绞痛) AND (中医 OR 中药 OR 中医药 OR 草药 OR 中草药 OR 方剂 OR 药方 OR 颗粒 OR 中成药) AND (血脂 OR 脂质代谢 OR 脂质 OR 低密度脂蛋白 OR 脂蛋白 OR LDL-C OR 胆固醇 OR 甘油三酯 OR 高密度脂蛋白 OR HDL-C) AND (随机对照试验 OR 随机对照研究 OR RCT OR 随机 OR 临床研究)
