# Supplementary material for: Traditional Chinese medicine lowering lipid levels and cardiovascular events across baseline lipid levels among coronary heart disease: a meta-analysis of randomized controlled trials
Source: Front Cardiovasc Med. 2024 Jul 11;11:1407536. doi: 10.3389/fcvm.2024.1407536 (PMC11269158; doi:10.3389/fcvm.2024.1407536)
Supplement: Supplementary file 2 [file Table2.docx]

**Supplementary material S2a Risk of bias of included RCTs**

| **Study (year)** | **Random sequence generation** | **Allocation concealment** | **Blinding of participants and personnel** | **Blinding of outcome assessment** | **Incomplete outcome data** | **Selective reporting** | **Other bias** |
| --- | --- | --- | --- | --- | --- | --- | --- |
| Xu, 2018 | L | U | H | U | L | L | U |
| Li, 2016 | L | U | H | U | L | L | U |
| Yang, 2016 | L | U | H | U | L | L | U |
| Zhou, 2021 | U | U | H | U | L | L | U |
| Tang, 2021 | L | U | H | U | L | L | U |
| Qin, 2021 | L | U | H | U | L | L | U |
| Tang, 2016 | L | U | H | U | L | L | U |
| Cheng, 2010 | L | U | H | L | L | H | U |
| Ma, 2015 | U | U | H | U | L | L | U |
| Zhang, 2015 | L | U | H | U | L | L | U |
| Li, 2018 | L | U | H | U | L | L | U |
| Chen, 2008 | U | U | H | U | L | L | U |
| Lin, 2011 | U | U | H | U | L | L | U |
| Zhao, 2004 | U | U | H | U | L | H | U |
| Kong, 2022 | L | U | H | U | L | L | U |
| Dai, 2011 | U | U | H | U | L | L | U |
| Tan, 2022 | L | L | H | U | L | L | U |
| Lu, 2008 | U | L | L | U | L | L | U |
| Li, 2011 | L | U | H | U | L | L | U |
| Sun, 2011 | U | U | H | U | L | H | U |
| Li, 2013 | U | U | H | U | L | L | U |
| Li, 2012 | U | U | H | U | L | L | U |
| Zhao, 2009 | L | U | H | U | L | H | U |

**Supplementary material S2b. GRADE for quality assessment**

| **Certainty assessment** | | | | | | | **№ of patients** | | **Effect** | | **Certainty** | **Importance** |
| --- | --- | --- | --- | --- | --- | --- | --- | --- | --- | --- | --- | --- |
| **№ of studies** | **Study design** | **Risk of bias** | **Inconsistency** | **Indirectness** | **Imprecision** | **Other considerations** | **CHM** | **WM** | **Relative (95% CI)** | **Absolute (95% CI)** |  |  |
| **CHM for LDL-C level by baseline LDL-C level** | | | | | | | | | | | | |
| 23 | randomised trials | serious^a^ | serious^b^ | not serious | not serious | none | 3670 | 3646 | - | MD **0.46 lower** (0.6 lower to 0.32 lower) | ⨁⨁◯◯ Low | CRITICAL |
| **CHM for LDL-C level by baseline TG level** | | | | | | | | | | | | |
| 20 | randomised trials | serious^a^ | serious^b^ | not serious | not serious | none | 3398 | 3383 | - | MD **0.27 lower** (0.34 lower to 0.2 lower) | ⨁⨁◯◯ Low | CRITICAL |
| **CHM for LDL-C level by baseline TC level** | | | | | | | | | | | | |
| 20 | randomised trials | serious^a^ | serious^b^ | not serious | not serious | none | 3398 | 3383 | - | MD **0.76 lower** (0.97 lower to 0.54 lower) | ⨁⨁◯◯ Low | CRITICAL |
| **CHM for LDL-C level by baseline HDL-C level** | | | | | | | | | | | | |
| 17 | randomised trials | serious^a^ | serious^a^ | not serious | not serious | none | 3219 | 3204 | - | MD **0.12 higher** (0.07 higher to 0.18 higher) | ⨁⨁◯◯ Low | CRITICAL |
| **CHM for MACEs stratified by baseline LDL-C level** | | | | | | | | | | | | |
| 23 | randomised trials | serious^a^ | not serious | not serious | not serious | publication bias strongly suspected^c^ | 424/3670 (11.6%) | 825/3646 (22.6%) | **RR 0.51** (0.46 to 0.57) | **111 fewer per 1,000** (from 122 fewer to 97 fewer) | ⨁⨁◯◯ Low | CRITICAL |
| **CHM for MACEs stratified by baseline TG level** | | | | | | | | | | | | |
| 20 | randomised trials | serious^a^ | not serious | not serious | not serious | publication bias strongly suspected^c^ | 381/3398 (11.2%) | 740/3383 (21.9%) | **RR 0.51** (0.46 to 0.57) | **107 fewer per 1,000** (from 118 fewer to 94 fewer) | ⨁⨁◯◯ Low | CRITICAL |
| **CHM for MACEs stratified by baseline TC level** | | | | | | | | | | | | |
| 20 | randomised trials | serious^a^ | not serious | not serious | not serious | publication bias strongly suspected^c^ | 381/3398 (11.2%) | 740/3383 (21.9%) | **RR 0.51** (0.46 to 0.57) | **107 fewer per 1,000** (from 118 fewer to 94 fewer) | ⨁⨁◯◯ Low | CRITICAL |
| **CHM for MACEs stratified by baseline HDL-C level** | | | | | | | | | | | | |
| 17 | randomised trials | serious^a^ | not serious | not serious | not serious | publication bias strongly suspected^c^ | 359/3219 (11.2%) | 686/3204 (21.4%) | **RR 0.52** (0.47 to 0.58) | **103 fewer per 1,000** (from 113 fewer to 90 fewer) | ⨁⨁◯◯ Low | CRITICAL |

**CI:** confidence interval; **MD:** mean difference; **RR:** risk ratio

#### Explanations

a. Downgrading one level for the randomization method.

b. Downgrading one level for the heterogeneity ≥ 50%.

c. Downgrading one level for potential publication bias.
