# Supplementary material for: Traditional Chinese medicine lowering lipid levels and cardiovascular events across baseline lipid levels among coronary heart disease: a meta-analysis of randomized controlled trials
Source: Front Cardiovasc Med. 2024 Jul 11;11:1407536. doi: 10.3389/fcvm.2024.1407536 (PMC11269158; doi:10.3389/fcvm.2024.1407536)
Supplement: Supplementary file 3 [file Table3.docx]

# Supplementary material S3. Sources of Statistical Heterogeneity in Subgroup Analyses

| **Subgroup** | | **Number of studies** | **Number of patients** | | **WT** | **MD [CI 95%]** | ***I^2^*** | ***P*** | ***P* value for interaction** |
| --- | --- | --- | --- | --- | --- | --- | --- | --- | --- |
|  |  |  | **Trail** | **Control** |  |  |  |  |  |
| **Analysis by baseline LDL-C** | | | | | | | | | |
| Age | age<60 | 7 | 2745 | 2758 | 36.2% | -0.40 [-0.57, -0.24] | 94% | P<0.00001 | *P* = 0.36 |
|  | age≥60 | 13 | 767 | 752 | 63.8% | -0.55 [-0.83, -0.27] | 97% | P<0.0001 |  |
|  | overall | 20 | 3512 | 3510 | 100.0% | -0.50 [-0.65, -0.35] | 96% | P<0.00001 |  |
| Statins | used statins | 16 | 1004 | 985 | 68.6% | -0.51 [-0.73, -0.28] | 97% | P<0.0001 | *P* = 0.30 |
|  | not used statins | 7 | 2666 | 2661 | 31.4% | -0.35 [-0.54, -0.16] | 96% | P=0.0002 |  |
|  | overall | 23 | 3670 | 3646 | 100.0% | -0.46 [-0.60, -0.32] | 96% | P<0.00001 |  |
| Course of treatment | <6 months | 17 | 972 | 935 | 75.5% | -0.48 [-0.66, -0.30] | 97% | P<0.00001 | *P* = 0.57 |
|  | 6-12 months | 3 | 106 | 105 | 11.2% | -0.51 [-0.77, -0.25] | 61% | P=0.0001 |  |
|  | ≥12 months | 3 | 2592 | 2606 | 13.4% | -0.26 [-0.67, 0.14] | 95% | P=0.20 |  |
|  | overall | 23 | 3670 | 3646 | 100.0% | -0.46 [-0.60, -0.32] | 96% | P<0.00001 |  |
| Type of Disease | Post-PCI | 7 | 344 | 340 | 30.9% | -0.41 [-0.51, -0.30] | 65% | P<0.00001 | *P* = 0.60 |
|  | CCS | 5 | 2769 | 2777 | 22.2% | -0.34 [-0.61, -0.07] | 93% | P=0.01 |  |
|  | ACS | 11 | 557 | 529 | 46.9% | -0.54 [-0.85, -0.24] | 98% | P=0.0005 |  |
|  | overall | 23 | 3670 | 3646 | 100.0% | -0.46 [-0.60, -0.32] | 96% | P<0.00001 |  |
| **Analysis by baseline TG level** | | | | | | | | | |
| Age | age<60 | 7 | 2745 | 2758 | 42.9% | -0.31 [-0.44, -0.18] | 91% | P<0.00001 | *P* = 0.53 |
|  | age≥60 | 10 | 495 | 489 | 57.1% | -0.26 [-0.35, -0.17] | 69% | P<0.00001 |  |
|  | overall | 17 | 3240 | 3247 | 100.0% | -0.28 [-0.35, -0.20] | 84% | P<0.00001 |  |
| Statins | used statins | 13 | 732 | 722 | 65.3% | -0.24 [-0.34, -0.14] | 80% | P<0.00001 | *P* = 0.23 |
|  | not used statins | 7 | 2666 | 2661 | 34.7% | -0.33 [-0.45, -0.21] | 96% | P<0.00001 |  |
|  | overall | 20 | 3398 | 3383 | 100.0% | -0.27 [-0.34, -0.20] | 81% | P<0.00001 |  |
| Course of treatment | <6 months | 16 | 854 | 825 | 82.0% | -0.27 [-0.34, -0.19] | 77% | P<0.00001 | *P* = 0.0003 |
|  | 6-12 months | 2 | 72 | 72 | 8.0% | -0.50 [-0.64, -0.36] | 0% | P<0.00001 |  |
|  | ≥12 months | 2 | 2472 | 2486 | 10.0% | -0.16 [-0.25, -0.07] | 9% | P=0.0006 |  |
|  | overall | 20 | 3398 | 3383 | 100.0% | -0.27 [-0.34, -0.20] | 81% | P<0.00001 |  |
| Type of Disease | Post-PCI | 7 | 344 | 340 | 35.1% | -0.30 [-0.42, -0.19] | 74% | P<0.00001 | *P* = 0.43 |
|  | CCS | 4 | 2649 | 2657 | 23.9% | -0.21 [-0.30, -0.12] | 48% | P<0.00001 |  |
|  | ACS | 9 | 405 | 386 | 41.0% | -0.26 [-0.40, -0.12] | 83% | P=0.0003 |  |
|  | overall | 20 | 3398 | 3383 | 100.0% | -0.27 [-0.34, -0.20] | 81% | P<0.00001 |  |
| **Analysis by baseline TC level** | | | | | | | | | |
| Age | age<60 | 7 | 2745 | 2758 | 41.0% | -0.63 [-0.79, -0.48] | 74% | P<0.00001 | *P* = 0.32 |
|  | age≥60 | 10 | 495 | 489 | 59.0% | -0.87 [-1.30, -0.44] | 96% | P<0.0001 |  |
|  | overall | 17 | 3240 | 3247 | 100.0% | -0.78 [-1.02, -0.54] | 96% | P<0.00001 |  |
| Statins | used statins | 13 | 732 | 722 | 65.3% | -0.76 [-1.12, -0.41] | 96% | P<0.0001 | *P* = 0.84 |
|  | not used statins | 7 | 2666 | 2661 | 34.7% | -0.72 [-0.90, -0.55] | 73% | P<0.00001 |  |
|  | overall | 20 | 3398 | 3383 | 100.0% | -0.76 [-0.97, -0.54] | 95% | P<0.00001 |  |
| Course of treatment | <6 months | 16 | 854 | 825 | 79.6% | -0.78 [-1.06, -0.49] | 95% | P<0.00001 | *P* = 0.03 |
|  | 6-12 months | 2 | 72 | 72 | 9.6% | -1.11 [-1.39, -0.83] | 0% | P<0.00001 |  |
|  | ≥12 months | 2 | 2472 | 2486 | 10.8% | -0.28 [-0.89, 0.34] | 93% | P=0.38 |  |
|  | overall | 20 | 3398 | 3383 | 100.0% | -0.76 [-0.97, -0.54] | 95% | P<0.00001 |  |
| Type of Disease | Post-PCI | 7 | 344 | 340 | 35.1% | -0.77 [-1.25, -0.29] | 97% | P=0.002 | *P* = 0.37 |
|  | CCS | 4 | 2649 | 2657 | 21.1% | -0.55 [-0.77, -0.34] | 75% | P<0.00001 |  |
|  | ACS | 9 | 405 | 386 | 43.8% | -0.85 [-1.24, -0.46] | 92% | P<0.0001 |  |
|  | overall | 20 | 3398 | 3383 | 100.0% | -0.76 [-0.97, -0.54] | 95% | P<0.00001 |  |
| **Analysis by baseline HDL-C level** | | | | | | | | | |
| Age | age<60 | 6 | 2670 | 2683 | 44.4% | 0.11 [0.05, 0.17] | 83% | P=0.0003 | *P* = 0.52 |
|  | age≥60 | 8 | 391 | 385 | 55.6% | 0.15 [0.04, 0.25] | 85% | P=0.005 |  |
|  | overall | 14 | 3061 | 3068 | 100.0% | 0.14 [0.08, 0.20] | 92% | P<0.00001 |  |
| Statins | used statins | 10 | 553 | 543 | 52.7% | 0.11 [0.03, 0.19] | 76% | P=0.006 | *P* = 0.67 |
|  | not used statins | 7 | 2666 | 2661 | 47.3% | 0.13 [0.06, 0.20] | 90% | P=0.0002 |  |
|  | overall | 17 | 3219 | 3204 | 100.0% | 0.12 [0.07, 0.18] | 91% | P<0.0001 |  |
| Course of treatment | <6 months | 13 | 675 | 646 | 71.6% | 0.09 [0.03, 0.15] | 84% | P=0.004 | *P* < 0.0001 |
|  | 6-12 months | 2 | 72 | 72 | 12.6% | 0.43 [0.26, 0.60] | 69% | P<0.00001 |  |
|  | ≥12 months | 2 | 2472 | 2486 | 15.8% | 0.05 [0.03, 0.07] | 0% | P<0.00001 |  |
|  | overall | 17 | 3219 | 3204 | 100.0% | 0.12 [0.07, 0.18] | 91% | P<0.0001 |  |
| Type of Disease | Post-PCI | 7 | 344 | 340 | 41.6% | 0.19 [0.08, 0.30] | 87% | P=0.0008 | *P* = 0.11 |
|  | CCS | 4 | 2649 | 2657 | 26.7% | 0.09 [0.00, 0.18] | 68% | P=0.05 |  |
|  | ACS | 6 | 226 | 207 | 31.7% | 0.05 [-0.00, 0.11] | 47% | P=0.06 |  |
|  | overall | 17 | 3219 | 3204 | 100.0% | 0.12 [0.07, 0.18] | 91% | P<0.0001 |  |
