# Supplementary material for: Traditional Chinese medicine lowering lipid levels and cardiovascular events across baseline lipid levels among coronary heart disease: a meta-analysis of randomized controlled trials
Source: Front Cardiovasc Med. 2024 Jul 11;11:1407536. doi: 10.3389/fcvm.2024.1407536 (PMC11269158; doi:10.3389/fcvm.2024.1407536)
Supplement: Supplementary file 4 [file Table4.docx]

# Supplementary material S4. Sensitivity analysis of CHM for lipid level after deletion of individual studies

| **Study excluded** | **Mean Difference[95%CI]** | | | |
| --- | --- | --- | --- | --- |
|  | **LDL-C** | **TG** | **TC** | **HDL-C** |
| Xu, 2018 | -0.46 [-0.60, -0.31] | -0.28 [-0.35, -0.20] | -0.77 [-1.00, -0.53] | 0.13 [0.07, 0.18] |
| Li, 2016 | -0.45 [-0.59, -0.31] | / | / | / |
| Yang, 2016 | -0.48 [-0.62, -0.33] | -0.28 [-0.35, -0.21] | -0.78 [-1.01, -0.55] | 0.13 [0.07, 0.19] |
| Zhou, 2021 | -0.47 [-0.62, -0.31] | -0.26 [-0.34, -0.19] | -0.70 [-0.86, -0.54] | 0.11 [0.06, 0.17] |
| Tang, 2021 | -0.46 [-0.61, -0.32] | -0.26 [-0.32, -0.19] | -0.74 [-0.96, -0.51] | 0.10 [0.05, 0.15] |
| Qin, 2021 | -0.45 [-0.60, -0.31] | -0.28 [-0.35, -0.21] | -0.79 [-1.02, -0.57] | 0.13 [0.07, 0.18] |
| Tang, 2016 | -0.47 [-0.61, -0.33] | -0.24 [-0.31, -0.18] | -0.74 [-0.96, -0.51] | / |
| Cheng, 2010 | -0.45 [-0.59, -0.31] | -0.27 [-0.34, -0.20] | -0.74 [-0.96, -0.51] | 0.11 [0.05, 0.16] |
| Ma, 2015 | -0.46 [-0.60, -0.31] | -0.27 [-0.34, -0.20] | -0.74 [-0.97, -0.52] | / |
| Zhang, 2015 | -0.47 [-0.63, -0.32] | -0.26 [-0.34, -0.19] | -0.77 [-1.00, -0.54] | 0.13 [0.06, 0.19] |
| Li, 2018 | -0.45 [-0.59, -0.31] | -0.26 [-0.34, -0.19] | -0.75 [-0.97, -0.52] | 0.11 [0.06, 0.17] |
| Chen, 2008 | -0.47 [-0.61, -0.32] | -0.27 [-0.34, -0.20] | -0.77 [-0.99, -0.54] | 0.12 [0.07, 0.18] |
| Lin, 2011 | -0.45 [-0.59, -0.31] | / | / | / |
| Zhao, 2004 | -0.46 [-0.61, -0.32] | -0.28 [-0.35, -0.21] | -0.79 [-1.01, -0.56] | 0.12 [0.07, 0.18] |
| Kong, 2022 | -0.46 [-0.60, -0.32] | -0.26 [-0.34, -0.19] | -0.76 [-0.99, -0.54] | 0.13 [0.07, 0.18] |
| Dai, 2011 | -0.45 [-0.59, -0.31] | -0.28 [-0.35, -0.21] | -0.70 [-0.92, -0.48] | 0.13 [0.07, 0.18] |
| Tan, 2022 | -0.46 [-0.61, -0.32] | -0.27 [-0.34, -0.20] | -0.78 [-1.00, -0.55] | 0.13 [0.07, 0.19] |
| Lu, 2008 | -0.45 [-0.61, -0.30] | -0.28 [-0.35, -0.21] | -0.77 [-1.03, -0.50] | 0.13 [0.07, 0.19] |
| Li, 2011 | -0.37 [-0.46, -0.28] | -0.27 [-0.34, -0.20] | -0.71 [-0.93, -0.49] | / |
| Sun, 2011 | -0.47 [-0.61, -0.33] | -0.28 [-0.35, -0.21] | -0.80 [-1.02, -0.58] | 0.13 [0.07, 0.18] |
| Li, 2013 | -0.47 [-0.61, -0.32] | -0.27 [-0.34, -0.20] | -0.78 [-1.00, -0.55] | 0.13 [0.07, 0.18] |
| Li, 2012 | -0.48 [-0.62, -0.34] | / | / | / |
| Zhao, 2009 | -0.48 [-0.62, -0.34] | -0.27 [-0.34, -0.20] | -0.74 [-0.97, -0.52] | 0.13 [0.08, 0.19] |
