# Supplementary material for: Traditional Chinese medicine lowering lipid levels and cardiovascular events across baseline lipid levels among coronary heart disease: a meta-analysis of randomized controlled trials
Source: Front Cardiovasc Med. 2024 Jul 11;11:1407536. doi: 10.3389/fcvm.2024.1407536 (PMC11269158; doi:10.3389/fcvm.2024.1407536)
Supplement: Supplementary file 5 [file Table5.docx]

# Supplementary material S5. Meta-analysis of CHM for MACEs Stratified by magnitude of lipid reduction

| **Subgroup** | | **No. of studies** | **No. of patients with events/total** | | **WT** | **RR [CI 95%]** | **I^2^** | ***P*** | **P value for interaction** |
| --- | --- | --- | --- | --- | --- | --- | --- | --- | --- |
|  |  |  | **T** | **C** |  |  |  |  |  |
| **Analysis by LDL-C level** | | | | | | | | | |
| Magnitude of LDL-C reduction | <1 mmol/L | 15 | 323/3266 | 603/3250 | 84.0% | 0.53 [0.47, 0.60] | 0% | P<0.00001 | P = 0.34 |
|  | 1-2 mmol/L | 4 | 14/142 | 39/141 | 5.5% | 0.35 [0.21, 0.60] | 8% | P=0.0001 |  |
|  | ≥2 mmol/L | 4 | 40/262 | 75/255 | 10.5% | 0.52 [0.38, 0.72] | 0% | P<0.0001 |  |
|  | overall | 23 | 377/3670 | 717/3646 | 100.0% | 0.52 [0.47, 0.58] | 0% | P<0.00001 |  |
| **Analysis by TG level** | | | | | | | | | |
| Magnitude of TG reduction | <0.5 mmol/L | 8 | 300/2896 | 536/2906 | 82.8% | 0.56 [0.49, 0.64] | 0% | P<0.00001 | P = 0.02 |
|  | 0.5-1.0 mmol/L | 8 | 33/317 | 80/292 | 12.7% | 0.39 [0.27, 0.56] | 0% | P<0.00001 |  |
|  | ≥1.0 mmol/L | 4 | 6/185 | 29/185 | 4.5% | 0.21 [0.09, 0.49] | 0% | P=0.0003 |  |
|  | overall | 20 | 339/3398 | 645/3383 | 100.0% | 0.52 [0.47, 0.59] | 0% | P<0.00001 |  |
| **Analysis by TC level** | | | | | | | | | |
| Baseline TC level | <5.2 mmol/L | 7 | 68/374 | 126/351 | 19.7% | 0.53 [0.42, 0.67] | 0% | P<0.00001 | P = 0.24 |
|  | 5.2-6.2 mmol/L | 8 | 253/2808 | 467/2815 | 72.3% | 0.54 [0.47, 0.63] | 43% | P<0.00001 |  |
|  | ≥6.2 mmol/L | 5 | 18/216 | 52/217 | 8.0% | 0.35 [0.21, 0.57] | 0% | P<0.0001 |  |
|  | overall | 21 | 339/3398 | 645/3383 | 100.0% | 0.52 [0.47, 0.59] | 0% | P<0.00001 |  |
| Magnitude of TC reduction | <1 mmol/L | 5 | 274/2692 | 479/2702 | 74.0% | 0.57 [0.50, 0.66] | 0% | P<0.00001 | P = 0.02 |
|  | 1-2 mmol/L | 8 | 39/364 | 90/343 | 14.2% | 0.41 [0.30, 0.58] | 22% | P<0.00001 |  |
|  | ≥2 mmol/L | 7 | 26/342 | 76/338 | 11.9% | 0.34 [0.22, 0.51] | 0% | P<0.00001 |  |
|  | overall | 20 | 339/3398 | 645/3383 | 100.0% | 0.52 [0.47, 0.59] | 0% | P<0.00001 |  |
| **Analysis by HDL-C level** | | | | | | | | | |
| Baseline HDL-C | <1.03 mmol/L | 5 | 11/205 | 37/182 | 6.6% | 0.26 [0.14, 0.50] | 0% | P<0.0001 | P = 0.05 |
|  | 1.03-1.55 mmol/L | 7 | 283/2832 | 503/2842 | 84.8% | 0.57 [0.50, 0.65] | 0% | P<0.00001 |  |
|  | ≥1.55 mmol/L | 5 | 23/182 | 51/180 | 8.7% | 0.44 [0.29, 0.69] | 0% | P=0.0003 |  |
|  | overall | 17 | 317/3219 | 591/3204 | 100.0% | 0.53 [0.47, 0.61] | 0% | P<0.00001 |  |
| Magnitude of HDL-C reduction | <0.5 mmol/L | 14 | 306/3110 | 567/3098 | 95.9% | 0.54 [0.48, 0.61] | 0% | P<0.00001 | P = 0.51 |
|  | >0.5 mmol/L | 3 | 11/109 | 24/106 | 4.1% | 0.43 [0.23, 0.83] | 10% | P=0.01 |  |
|  | overall | 17 | 317/3219 | 591/3204 | 100.0% | 0.53 [0.47, 0.61] | 0% | P<0.00001 |  |
