# Supplementary material for: Traditional Chinese medicine lowering lipid levels and cardiovascular events across baseline lipid levels among coronary heart disease: a meta-analysis of randomized controlled trials
Source: Front Cardiovasc Med. 2024 Jul 11;11:1407536. doi: 10.3389/fcvm.2024.1407536 (PMC11269158; doi:10.3389/fcvm.2024.1407536)
Supplement: Supplementary file 7 [file Table7.docx]

# Supplementary material S7. Meta-analysis of CHM for each MACEs Stratified by baseline lipid level

| **Subgroup** | | **Risk Ratio [95% CI]** | | | | | |
| --- | --- | --- | --- | --- | --- | --- | --- |
|  |  | **Cardiovascular mortality** | **Myocardial infarction** | **Revascularization** | **Angina pectoris** | **Heart failure** | **Nonfatal stroke** |
| Baseline LDL-C level | <2.59 mmol/L | / | 0.50 [0.05, 5.43] | / | 0.38 [0.10, 1.37] | 0.63 [0.21, 1.84] | / |
|  | 2.59-3.34 mmol/L | 0.24 [0.06, 0.93] | 0.35 [0.18, 0.68] | 0.67 [0.12, 3.71] | 0.33 [0.20, 0.54] | 0.43 [0.16, 1.15] | 0.33 [0.01, 7.93] |
|  | 3.34-4.12 mmol/L | 0.70 [0.55, 0.89] | 0.43 [0.32, 0.58] | 0.64 [0.48, 0.86] | 0.41 [0.25, 0.65] | 0.57 [0.20, 1.64] | 0.47 [0.11, 2.02] |
|  | ≥4.12 mmol/L | 0.46 [0.18, 1.18] | 0.48 [0.26, 0.89] | 0.71 [0.31, 1.63] | 0.56 [0.33, 0.96] | / | 0.49 [0.20, 1.18] |
|  | overall | 0.65 [0.52, 0.82] | 0.43 [0.33, 0.54] | 0.65 [0.49, 0.85] | 0.40 [0.30, 0.54] | 0.52 [0.29, 0.95] | 0.47 [0.23, 0.98] |
|  | Overall effect P value | P=0.0003 | P<0.00001 | P=0.002 | P<0.00001 | P=0.03 | P=0.05 |
|  | Interaction P value | P = 0.23 | P = 0.92 | P=0.98 | P = 0.54 | P = 0.87 | P = 0.97 |
| Baseline TG level | <1.70 mmol/L | 0.67 [0.12, 3.71] | 0.80 [0.42, 1.50] | / | 0.48 [0.30, 0.75] | 0.68 [0.29, 1.61] | 0.52 [0.10, 2.71] |
|  | 1.70-2.25 mmol/L | 0.68 [0.53, 0.86] | 0.40 [0.29, 0.53] | 0.65 [0.49, 0.88] | 0.35 [0.20, 0.60] | 0.39 [0.12, 1.34] | / |
|  | ≥2.25 mmol/L | 0.37 [0.11, 1.24] | 0.43 [0.19, 1.00] | 0.34 [0.05, 2.08] | 0.21 [0.07, 0.61] | 0.44 [0.14, 1.41] | 0.25 [0.07, 0.86] |
|  | overall | 0.66 [0.52, 0.83] | 0.44 [0.34, 0.57] | 0.64 [0.48, 0.86] | 0.37 [0.27, 0.52] | 0.52 [0.29, 0.95] | 0.32 [0.12, 0.85] |
|  | Overall effect P value | P=0.0004 | P<0.00001 | P=0.003 | P<0.00001 | P=0.03 | P=0.02 |
|  | Interaction P value | P = 0.63 | P = 0.15 | P = 0.48 | P = 0.33 | P = 0.73 | P = 0.49 |
| Baseline TC level | <5.2 mmol/L | 0.19 [0.01, 3.90] | 0.61 [0.32, 1.19] | 0.67 [0.12, 3.71] | 0.45 [0.29, 0.70] | 0.68 [0.29, 1.61] | 0.52 [0.10, 2.71] |
|  | 5.2-6.2 mmol/L | 0.68 [0.54, 0.87] | 0.40 [0.30, 0.54] | 0.65 [0.48, 0.88] | 0.33 [0.19, 0.56] | 0.43 [0.16, 1.15] | 0.33 [0.01, 7.93] |
|  | ≥6.2 mmol/L | 0.40 [0.13, 1.25] | 0.57 [0.25, 1.34] | 0.34 [0.05, 2.08] | 0.20 [0.05, 0.89] | 0.40 [0.08, 1.99] | 0.24 [0.06, 0.92] |
|  | overall | 0.66 [0.52, 0.83] | 0.44 [0.34, 0.57] | 0.64 [0.48, 0.86] | 0.37 [0.27, 0.52] | 0.52 [0.29, 0.95] | 0.32 [0.12, 0.85] |
|  | Overall effect P value | P=0.0004 | P<0.00001 | P=0.003 | P<0.00001 | P=0.03 | P=0.02 |
|  | Interaction P value | P = 0.48 | P = 0.42 | P = 0.78 | P = 0.45 | P = 0.74 | P = 0.78 |
| Baseline HDL-C level | <1.03 mmol/L | 0.33 [0.01, 7.96] | 0.22 [0.05, 0.99] | 0.45 [0.11, 1.94] | 0.19 [0.06, 0.61] | 0.56 [0.14, 2.29] | / |
|  | 1.03-1.55 mmol/L | 0.69 [0.54, 0.87] | 0.44 [0.33, 0.58] | 0.65 [0.48, 0.88] | 0.43 [0.28, 0.64] | 0.59 [0.26, 1.35] | 0.52 [0.10, 2.71] |
|  | ≥1.55 mmol/L | 0.44 [0.12, 1.62] | 0.70 [0.28, 1.80] | 0.51 [0.05, 5.43] | 0.20 [0.02, 1.62] | 0.33 [0.07, 1.60] | 0.25 [0.07, 0.86] |
|  | overall | 0.67 [0.53, 0.85] | 0.44 [0.34, 0.58] | 0.64 [0.48, 0.86] | 0.37 [0.25, 0.54] | 0.53 [0.28, 1.00] | 0.32 [0.12, 0.85] |
|  | Overall effect P value | P=0.001 | P<0.00001 | P=0.003 | P<0.00001 | P=0.05 | P=0.02 |
|  | Interaction P value | P = 0.73 | P = 0.41 | P = 0.88 | P = 0.36 | P = 0.81 | P = 0.49 |
