# Supplementary material for: Traditional Chinese medicine lowering lipid levels and cardiovascular events across baseline lipid levels among coronary heart disease: a meta-analysis of randomized controlled trials
Source: Front Cardiovasc Med. 2024 Jul 11;11:1407536. doi: 10.3389/fcvm.2024.1407536 (PMC11269158; doi:10.3389/fcvm.2024.1407536)
Supplement: Supplementary file 8 [file Table8.docx]

# Supplementary material S8. Meta-analysis Stratified for the age, statin used, course of treatment, and type of disease

| **Subgroup** | | **No. of studies** | **No. of patients with events/total** | | **WT** | **RR [95%CI]** | ***I^2^*** | ***P*** | ***P* value for interaction** |
| --- | --- | --- | --- | --- | --- | --- | --- | --- | --- |
|  |  |  | T | C |  |  |  |  |  |
| age | age<60 | 7 | 244/2745 | 439/2758 | 64.3% | 0.56 [0.48, 0.65] | 0% | P<0.00001 | P = 0.16 |
|  | age≥60 | 13 | 115/767 | 241/752 | 35.7% | 0.47 [0.39, 0.57] | 8% | P<0.00001 |  |
|  | overall | 21 | 359/3512 | 680/3510 | 100.0% | 0.53 [0.47, 0.59] | 0% | P<0.00001 |  |
| Statin used | used | 16 | 138/1004 | 297/985 | 41.6% | 0.46 [0.39, 0.54] | 0% | P<0.00001 | P = 0.06 |
|  | not used | 7 | 239/2666 | 420/2661 | 58.4% | 0.57 [0.49, 0.66] | 0% | P<0.00001 |  |
|  | overall | 23 | 377/3670 | 717/3646 | 100.0% | 0.52 [0.47, 0.58] | 0% | P<0.00001 |  |
| Course of treatment | <6 month | 17 | 110/972 | 248/935 | 35.0% | 0.43 [0.35, 0.52] | 0% | P<0.00001 | P = 0.03 |
|  | 6-12 months | 3 | 12/106 | 29/105 | 4.1% | 0.40 [0.23, 0.72] | 0% | P=0.002 |  |
|  | ≥12 months | 3 | 255/2592 | 440/2606 | 60.9% | 0.58 [0.51, 0.67] | 0% | P<0.00001 |  |
|  | overall | 23 | 377/3670 | 717/3646 | 100.0% | 0.52 [0.47, 0.58] | 0% | P<0.00001 |  |
| Type of disease | Post-PCI | 7 | 32/344 | 84/340 | 11.8% | 0.38 [0.26, 0.54] | 0% | P<0.00001 | P = 0.10 |
|  | CCS | 5 | 256/2769 | 455/2777 | 63.2% | 0.56 [0.49, 0.65] | 0% | P<0.00001 |  |
|  | ACS | 11 | 89/557 | 178/529 | 25.0% | 0.49 [0.40, 0.60] | 0% | P<0.00001 |  |
|  | overall | 23 | 377/3670 | 717/3646 | 100.0% | 0.52 [0.47, 0.58] | 0% | P<0.00001 |  |
