# Supplementary material for: Traditional Chinese medicine lowering lipid levels and cardiovascular events across baseline lipid levels among coronary heart disease: a meta-analysis of randomized controlled trials
Source: Front Cardiovasc Med. 2024 Jul 11;11:1407536. doi: 10.3389/fcvm.2024.1407536 (PMC11269158; doi:10.3389/fcvm.2024.1407536)
Supplement: Supplementary file 9 [file Table9.docx]

# Supplementary material S9

# Funnel plots for different outcomes


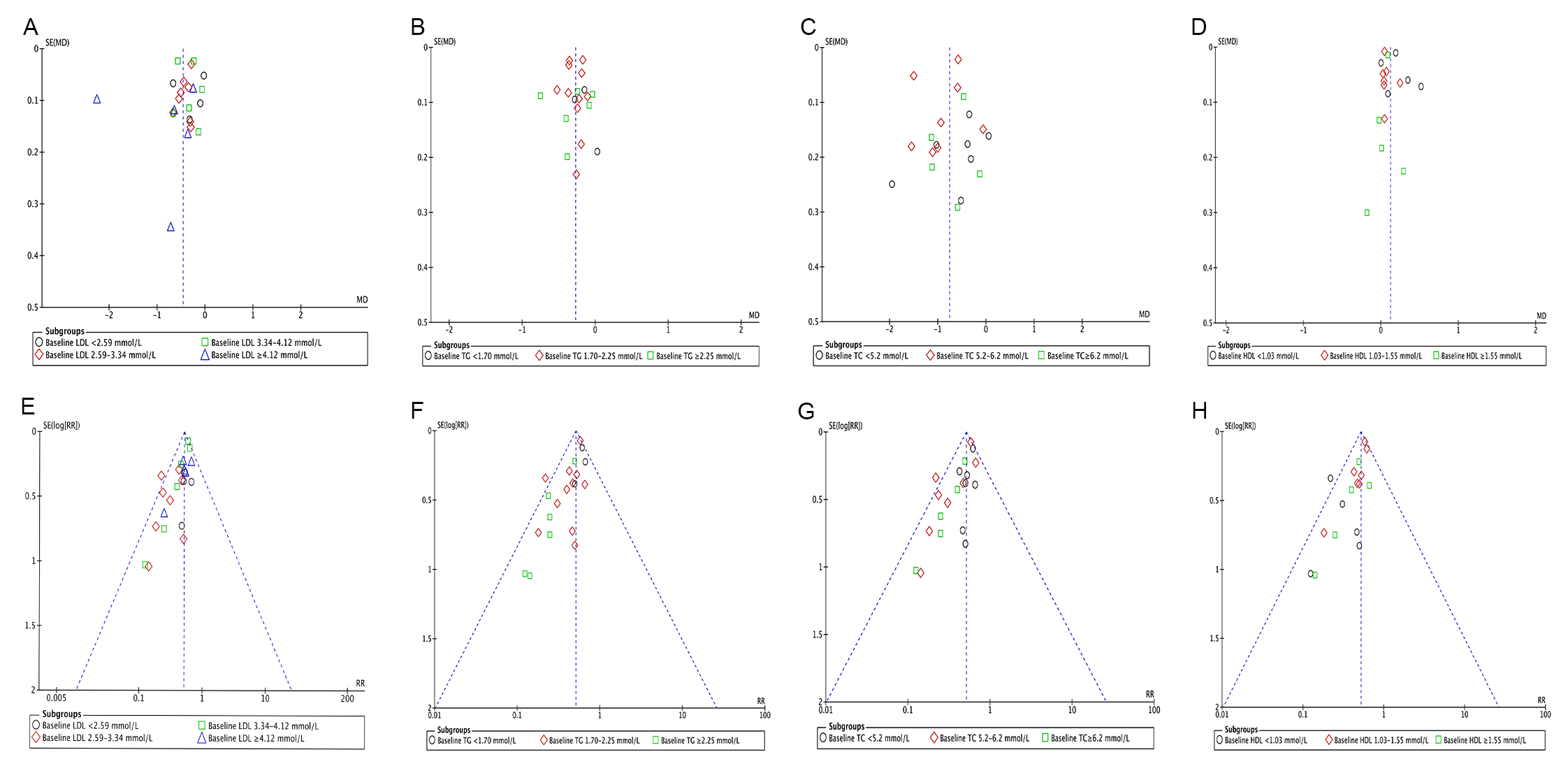


Supplementary material S9. Funnel plots for different outcomes. A: LDL-C level; B: TG level; C: TC level; D: HDL-C level; E: MACEs stratified by baseline LDL-C level; F: MACEs stratified by baseline TG level; G: MACEs stratified by baseline TC level; H: MACEs stratified by baseline HDL-C level.

**Egger’s test for different outcomes**

| **outcomes** | ***t* value** | ***P* value** | **95%*CI*** |
| --- | --- | --- | --- |
| LDL-C level | -0.73 | 0.473 | (-5.13, 2.46) |
| TG level | 0.11 | 0.913 | (-1.77, 1.97) |
| TC level | -0.56 | 0.583 | (-3.95, 2.29) |
| HDL-C level | 0.35 | 0.735 | (-1.98, 2.74) |
| MACEs stratified by baseline LDL-C level | -4.84 | 0.000 | (-1.70, -0.68) |
| MACEs stratified by baseline TG level | -4.58 | 0.000 | (-1.77, -0.66) |
| MACEs stratified by baseline TC level | -4.58 | 0.000 | (-1.77, -0.66) |
| MACEs stratified by baseline HDL-C level | -4.24 | 0.001 | (-1.72, -0.57) |
