# Supplementary material for: Traditional Chinese medicine lowering lipid levels and cardiovascular events across baseline lipid levels among coronary heart disease: a meta-analysis of randomized controlled trials
Source: Front Cardiovasc Med. 2024 Jul 11;11:1407536. doi: 10.3389/fcvm.2024.1407536 (PMC11269158; doi:10.3389/fcvm.2024.1407536)
Supplement: Supplementary file 10 [file Table10.docx]

# Supplementary material S10. Sensitivity analysis of CHM for MACEs after deletion of individual studies

| **Study excluded** | **Risk Ratio [95%CI]** | | | | | | |
| --- | --- | --- | --- | --- | --- | --- | --- |
|  | **MACEs** | **Cardiovascular mortality** | **Myocardial infarction** | **Angina pectoris** | **Revascularization** | **Heart failure** | **Nonfatal stroke** |
| Xu, 2018 | 0.52 [0.47, 0.59] | 0.66 [0.53, 0.84] | 0.42 [0.32, 0.54] | 0.41 [0.31, 0.54] | / | / | / |
| Li, 2016 | 0.52 [0.47, 0.59] | 0.66 [0.52, 0.83] | 0.44 [0.34, 0.57] | / | 0.64 [0.48, 0.85] | / | 0.32 [0.12, 0.85] |
| Yang, 2016 | 0.52 [0.47, 0.59] | / | 0.43 [0.33, 0.54] | 0.41 [0.30, 0.54] | / | 0.49 [0.24, 1.00] | / |
| Zhou, 2021 | 0.53 [0.47, 0.60] | / | 0.43 [0.34, 0.55] | 0.43 [0.32, 0.58] | / | 0.51 [0.27, 0.97] | / |
| Tang, 2021 | 0.52 [0.47, 0.59] | 0.66 [0.52, 0.83] | 0.43 [0.33, 0.55] | 0.40 [0.30, 0.54] | / | / | / |
| Qin, 2021 | 0.53 [0.47, 0.59] | / | 0.43 [0.33, 0.55] | / | / | 0.55 [0.29, 1.02] | 0.48 [0.23, 1.03] |
| Tang, 2016 | 0.53 [0.47, 0.59] | 0.66 [0.52, 0.83] | 0.43 [0.34, 0.55] | 0.41 [0.31, 0.55] | / | / | / |
| Cheng, 2010 | 0.53 [0.47, 0.59] | / | 0.43 [0.33, 0.55] | / | 0.66 [0.50, 0.87] | 0.53 [0.28, 0.98] | / |
| Ma, 2015 | 0.53 [0.47, 0.60] | 0.66 [0.53, 0.83] | 0.43 [0.34, 0.56] | 0.42 [0.31, 0.56] | / | 0.53 [0.28, 1.00] | / |
| Zhang, 2015 | 0.53 [0.47, 0.59] | 0.66 [0.52, 0.83] | 0.43 [0.33, 0.55] | / | / | 0.55 [0.29, 1.02] | / |
| Li, 2018 | 0.53 [0.47, 0.59] | / | 0.43 [0.33, 0.55] | 0.41 [0.31, 0.55] | / | 0.55 [0.30, 1.02] | / |
| Chen, 2008 | 0.52 [0.47, 0.59] | / | 0.43 [0.33, 0.55] | / | 0.65 [0.49, 0.85] | / | / |
| Lin, 2011 | 0.52 [0.47, 0.59] | 0.66 [0.52, 0.83] | 0.43 [0.33, 0.55] | 0.38 [0.28, 0.52] | / | / | / |
| Zhao, 2004 | 0.53 [0.47, 0.59] | / | 0.43 [0.33, 0.54] | 0.41 [0.31, 0.55] | / | / | 0.48 [0.23, 1.03] |
| Kong, 2022 | 0.52 [0.47, 0.59] | 0.66 [0.52, 0.83] | 0.41 [0.32, 0.53] | / | 0.65 [0.49, 0.86] | / | 0.65 [0.27, 1.57] |
| Dai, 2011 | 0.52 [0.46, 0.58] | / | / | / | / | / | / |
| Tan, 2022 | 0.52 [0.47, 0.59] | / | / | / | / | / | / |
| Lu, 2008 | 0.45 [0.38, 0.53] | 0.36 [0.17, 0.74] | 0.47 [0.33, 0.68] | / | 0.62 [0.33, 1.18] | / | / |
| Li, 2011 | 0.52 [0.46, 0.58] | 0.65 [0.52, 0.83] | 0.42 [0.32, 0.54] | 0.38 [0.28, 0.52] | / | / | / |
| Sun, 2011 | 0.52 [0.46, 0.58] | / | 0.40 [0.31, 0.52] | 0.40 [0.29, 0.55] | / | 0.48 [0.25, 0.94] | 0.46 [0.20, 1.05] |
| Li, 2013 | 0.53 [0.47, 0.59] | 0.66 [0.53, 0.83] | 0.43 [0.33, 0.55] | 0.39 [0.29, 0.53] | / | / | / |
| Li, 2012 | 0.52 [0.47, 0.59] | / | 0.42 [0.33, 0.54] | 0.40 [0.30, 0.55] | 0.65 [0.49, 0.86] | / | / |
| Zhao, 2009 | 0.52 [0.47, 0.59] | / | / | / | / | / | / |
