# Supplementary figures and images for: Traditional Chinese medicine lowering lipid levels and cardiovascular events across baseline lipid levels among coronary heart disease: a meta-analysis of randomized controlled trials
Source: Front Cardiovasc Med. 2024 Jul 11;11:1407536. doi: 10.3389/fcvm.2024.1407536 (PMC11269158; doi:10.3389/fcvm.2024.1407536)

**Supplementary material S11. Forest plot of adverse events**


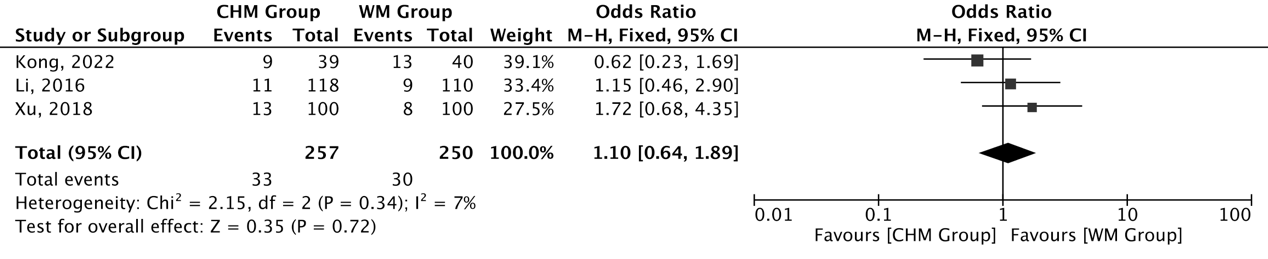

Supplement: Supplementary file 11 [file Table11.docx]
