# Supplementary material for: Traditional Chinese medicine lowering lipid levels and cardiovascular events across baseline lipid levels among coronary heart disease: a meta-analysis of randomized controlled trials
Source: Front Cardiovasc Med. 2024 Jul 11;11:1407536. doi: 10.3389/fcvm.2024.1407536 (PMC11269158; doi:10.3389/fcvm.2024.1407536)
Supplement: Supplementary file 12 [file Table12.docx]

**Supplementary material S12. The frequency statistics of CHM in included studies (Frequency ≥3)**

| Chinese name | Standardized name | Frequency |
| --- | --- | --- |
| Chuanxiong | Sichuan lovage rhizome (Rhizoma Ligustici Chuanxiong) | 13 |
| Danshen | Danshen root (Radix Salviae Miltiorrhizae) | 12 |
| Gualou | Snakegourd fruit (*Fructus Trichosanthis*) | 10 |
| Huangqi | Astragalus Radix (*Astragalus membranaceus (Fisch.) Bunge*) | 10 |
| Chishao | Peony root (Radix Paeoniae Rubra) | 9 |
| Zhigancao | Liquorice root (Radix Glycyrrhizae) | 8 |
| Danggui | Chinese angelica (Radix Angelicae Sinensis) | 8 |
| Guizhi | Cassia Twig (*Ramulus Cinnamomi*) | 8 |
| Renshen | Panax ginseng (*Radix Ginseng*) | 7 |
| Dangshen | Pilose Asiabell Root (Codonopsis pilosula) | 7 |
| Shanzha | Chinese Hawthorn (Crataegus pinnatifida Bunge) | 7 |
| Gegen | Root of lobed kudzuvine (Puerariae Lobatae Radix) | 6 |
| Sanqi | Sanchi (Panax notoginseng) | 6 |
| Honghua | Safflower (Flos Carthami) | 6 |
| Banxia | Pinellia tuber (Rhizoma Pinelliae), | 6 |
| Taoren | Peach Seed (Persicae Semen) | 4 |
| Fuling | Poria (Poria Cocos) | 4 |
| Chaihu | Root of Chinese Thorowax (Radix Bupleuri) | 4 |
| Xixin | Manchurian wildginger (Asari Radix Et Rhizoma) | 3 |
| Maidong | Dwarf Lilyturf Tuber (Ophiopogonis Radix) | 3 |
| Xiebai | Longstamen onion (Allii Macrostemonis Bulbus) | 3 |
| Yanhusuo | Corydalis Yanhusuo (Corydalis Rhizoma) | 3 |
| Chenpi | Dried tangerine peel (*Pericarpium Citri Reticulatae*) | 3 |
| Zhiqiao | fruit of Seville orange (Aurantii Fructus) | 3 |
| Shuizhi | Leech (Hirudo) | 3 |
| Yujin | Curcuma aromatica Salisb (Curcumae Radix) | 3 |
